# Contrasting Rare Earth Element Signatures Between Stromatolitic Carbonates and Lagoon Waters at Shark Bay, Western Australia: Implications for Paleo‐Environmental Reconstructions of Microbial Habitats

**DOI:** 10.1111/gbi.70055

**Published:** 2026-06-10

**Authors:** S. Viehmann, A.‐L. Zocher, S. V. Hohl, D. Kraemer, A. Martin, M. Markowska, S. Weyer

**Affiliations:** ^1^ Institute of Earth System Sciences, Section Mineralogy Leibniz University Hannover Hannover Germany; ^2^ Critical Metals for Enabling Technologies (CritMET), School of Science Constructor University Bremen Germany; ^3^ State Key Laboratory of Marine Geology Tongji University Shanghai People's Republic of China; ^4^ Federal Institute for Geosciences and Natural Resources (BGR) Hannover Germany; ^5^ Department of Geography and Environmental Sciences Northumbria University Newcastle UK

**Keywords:** carbonate, microbialite, REE, seawater, Shark Bay, stromatolite

## Abstract

Rare earth elements and yttrium (REY) signatures in stromatolitic carbonates have emerged as powerful geochemical proxies for reconstructing paleo‐depositional environments of microbial habitats. The applicability of such a proxy relies on the assumption that REY substitutes for calcium into crystal lattices, directly reflecting the composition of the fluid from which the carbonate precipitated. The REY signatures of stromatolites are often similar to those of open ocean seawater. In restricted environments, however, REY fractionation can occur between waters and stromatolites, questioning their reliability to reconstruct microbial habitats through Earth's history. In this contribution, we determined the REY concentrations and partition coefficients (Kd_(stromatolite‐fluid)_) of sub‐recent stromatolitic carbonates and ambient waters from the hypersaline Hamelin Pool in the Shark Bay lagoon, Australia. Shale‐normalized REY patterns of stromatolite morphologies show (except colloform morphologies) a middle REY_SN_ enrichment relative to the light and heavy REY_SN_. These signatures differ from those of seawater and ambient waters, suggesting that today's waters cannot be directly compared with stromatolitic carbonates from Shark Bay, which formed over thousands of years in a complex microbial mat system. Stromatolite morphologies such as colloform, smooth, or pustular structures formed in supratidal and intertidal environments exhibit the most variable Kd_(stromatolite‐fluid)_ values for the REY. The most dominant process affecting REY geochemistry in the Shark Bay stromatolites is most likely organic matter degradation and subsequent REY release into porewaters from which the carbonates formed in a (semi)closed microbial mat system. Cerebroid structures of the deepest lagoonal environment in the subtidal zone show the most constant Kd_(stromatolite‐fluid)_ values throughout the REY series, reflecting the least closed microbial mat system and a direct relationship between stromatolite morphology and water chemistry.

## 1 Introduction

Rare earth element and yttrium (REY) signatures in authigenic chemical sediments such as stromatolitic carbonates, i.e., ‘organo‐sedimentary structures formed by the incidental induction of mineral precipitation within or on microbial biofilms with, or without, trapping and binding of ambient sediments’ (Webb and Kamber 2000) emerged as a powerful geochemical tool to reconstruct paleo‐environments and physico‐chemical conditions prevalent in microbial habitats through Earth's history. Webb and Kamber (2000) reported shale‐normalized REY patterns (subscript SN; REY_SN_) of sub‐recent, Holocene microbialites from the Great Barrier Reef, Australia, that are parallel to open‐ocean seawater. These microbialites mainly consist of Mg‐calcite and show positive La_SN_, Gd_SN_, and Y_SN_ anomalies, a heavy REY_SN_ over light REY_SN_ enrichment, and redox‐related negative Ce_SN_ anomalies. These features are indicative of modern seawater (e.g., Alibo and Nozaki 1999). The similarities between microbial carbonate and the precipitating fluid have built a substantial theoretical basis for using stromatolitic carbonates as prime geochemical archives for reconstructing ancient depositional environments. Microbial habitats were reconstructed based on stromatolite geochemistry, extending back to the earliest appearance of stromatolites in the Paleoarchean Pilbara Craton (e.g., Van Kranendonk et al. 2002; Viehmann et al. 2020). In one of the many studies using REY in stromatolitic carbonates to reconstruct ancient depositional settings, Fogret et al. (2024) investigated REY signatures in the abundant microbialite collection at the Natural History Museum in Paris, spanning samples from the Archean to the present. Carbonate REY distributions were directly linked to depositional environments such as open marine, volcanic crater lakes, lagoonal, and freshwater settings (Fogret et al. 2024). These geochemical signatures reflect the wide range of microbialite‐forming environments in which microbial communities thrived and flourished during strikingly different atmospheric‐hydrospheric conditions through deep time (e.g., Dupraz and Visscher 2005; Howard and Sheldon 2025).

These reconstructions strongly rely on the assumption that the REY distribution of the fluids is directly mirrored by the carbonate that substituted the REY for calcium into the carbonate crystal lattice (Zhong and Mucci 1995). If this argument holds, there are additional influences that may impact REY geochemistry in carbonates, which have to be considered by using REY as geochemical proxies to reconstruct microbial habitats: (i) Syn‐depositional contamination by detrital aluminosilicates, phosphates, allochems, and/or Fe‐Mn oxides that are dissolved during the laboratory procedure (e.g., Schier et al. 2021), as well as post‐depositional REY mobility during fluid–rock interactions, may alter and mask the original REY signature of carbonates. Thus, the application of protocols to avoid syn‐ and post‐depositional alteration processes in stromatolitic carbonates is a prerequisite for using chemical sediments as archives to reconstruct paleo‐environments (cf. Kamber and Webb 2001; Viehmann et al. 2020, 2023). (ii) Methodological difficulties arise from the fact that sub‐recent stromatolitic carbonates sometimes contain REY concentrations as low as the lower ppb to ppt range, which are challenging to analyze with in situ laser ablation techniques and with solution techniques due to severe carbonate matrix effects and interferences on REY masses (e.g., Zhang et al. 2015). (iii) REY_SN_ patterns that are considered typical for open ocean seawater are also reported from freshwater settings such as groundwater (Johannesson et al. 1997), the Amazon distributary river waters (e.g., Merschel, Bau, Schmidt, et al. 2017), or even in microbialites of a Mexican lake system (Zeyen et al. 2021). These signatures reflect REY speciation and fractionation behavior in aqueous systems during sorption processes between REY solution complexes and organic/lithic surfaces, rather than being solely indicative of a marine origin (cf. Johannesson et al. (2014), for a review of REY behavior in aquatic systems). The dissolved loads of freshwater systems typically exhibit rather heterogeneous REY distributions that depend not only on REY speciation but also on the availability of particles and colloids (e.g., Elderfield et al. 1990). Thus, river waters are often dominated by organic (Merschel, Bau, Schmidt, et al. 2017) or lithic nanoparticles (Tepe and Bau 2016). Tepe and Bau (2016) simulated estuarine freshwater–seawater mixing processes and showed that the majority of REY is removed from solution at already less than 10% of seawater addition to create the typical seawater‐like REY_SN_ distributions.

Thus, caution must be exercised when using geochemical proxies, such as REY, in stromatolites and other carbonates without additional evidence from stratigraphic relationships to confirm depositional environmental conditions (Johannesson et al. 2006). Johannesson et al. (2014) also showed that REY distributions in stromatolites from non‐open‐ocean, but lacustrine, settings differ from ambient lake water. These authors proposed that REY are fractionated from the precipitating fluid in microbial environments before incorporation into the stromatolitic carbonate. The distinct heavy REY enrichment in the carbonate relative to ambient waters of the Cuatro Ciénegas bolson in Mexico was attributed to the preferential incorporation of heavy REY by organic ligands associated with cell walls, exopolymeric substances (EPS), and/or microbialite biofilms (Johannesson et al. 2014). However, the exact process(es) yielding fractionated REY signatures of stromatolitic carbonates in this restricted, non‐open ocean setting relative to the ambient fluid remain unclear. A better and more comprehensive understanding of REY behavior in stromatolites from restricted‐basin environments is urgently needed to improve the reliability of REY as a geochemical tool for paleo‐environmental reconstructions of microbial habitats across Earth's history.

To tackle this issue and further investigate REY partitioning and potential fractionation mechanisms between microbialites and ambient waters in non‐open‐ocean environments, we turned to one of the most intensively studied stromatolite environments on Earth: the hypersaline Hamelin Pool lagoon at Shark Bay in Western Australia. Shark Bay hosts stromatolite‐forming microbial habitats in subtidal, intertidal, and supratidal environments, representing unique natural laboratories for studying microbial habitats and providing blueprints for deep‐time settings and planetary studies. While the presence of different microbial communities (e.g., Babilonia et al. 2018; Reid et al. 2024; Suosaari et al. 2022; Vitek et al. 2022) has been a significant target of current and past research, a relatively complete geochemical characterization of the stromatolitic carbonates and ambient waters in the hypersaline lagoon is currently lacking. Here, we provide major and trace element data, as well as δ^13^C_carb_ and δ^18^O_carb_ values, for stromatolitic carbonates from supratidal, intertidal, and subtidal lagoon environments. These data are set in relation to REY data of ambient lagoon waters to investigate REY incorporation into stromatolitic carbonates. We applied REY preconcentration and matrix separation chemistry for carbonates with particularly low REY concentrations to bypass carbonate matrix effects and avoid interferences on REY masses during trace element analyses. The results allow us to identify potential REY fractionation processes in microbial habitats of extreme environments, such as hypersaline lagoons, and to assess their implications and reliability for the use of REY chemistry in deep‐time archives.

## 2 Geological Overview

**FIGURE 1:**
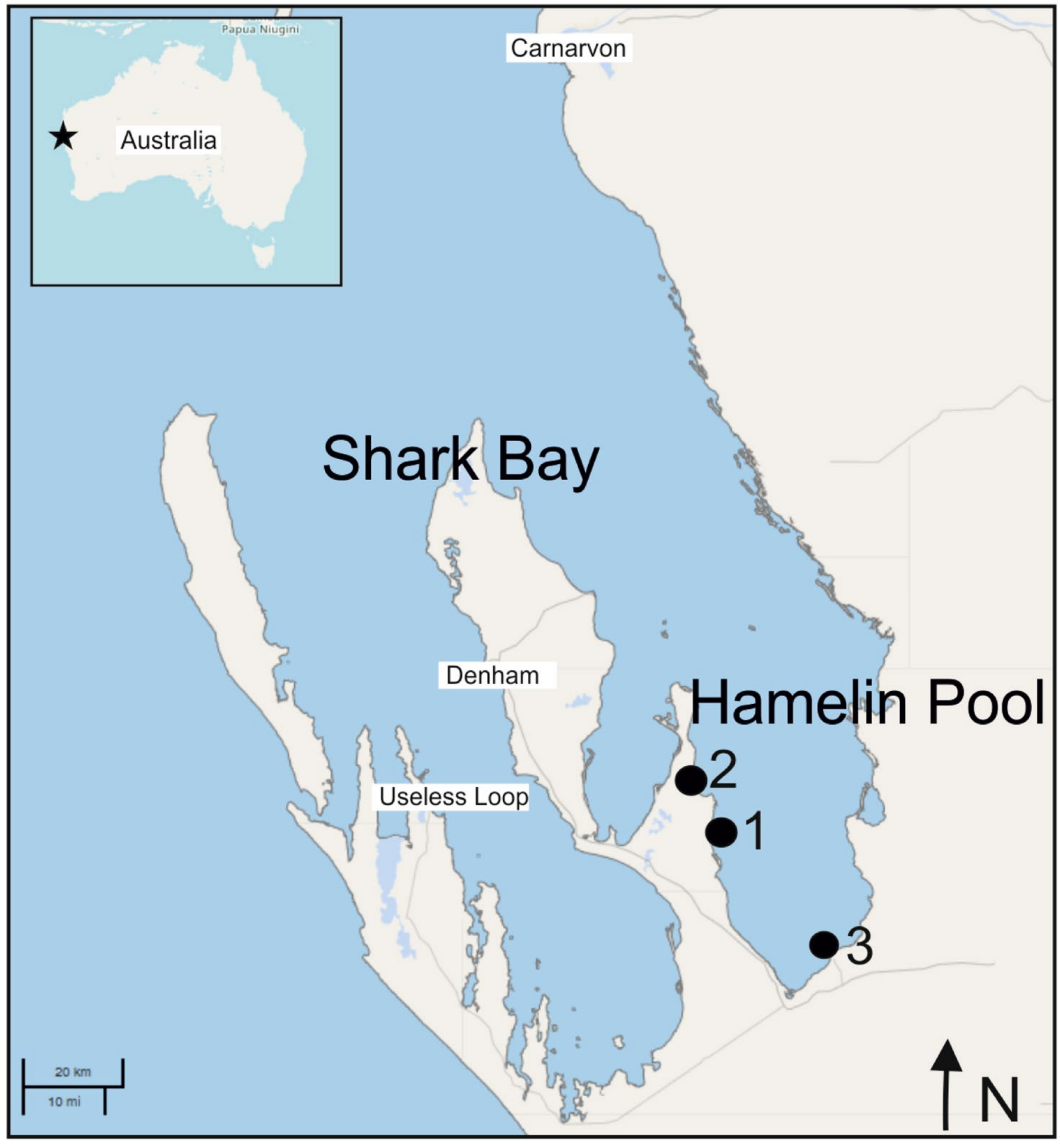
The location of the hypersaline Shark Bay lagoon in Western Australia. Stromatolitic carbonates and ambient water samples are collected from three locations (black circles) at Hamelin Pool, representing subtidal, intertidal, and supratidal settings (see Table 1 and Figure 2 for depositional environments). The black star in the inset shows the location of Shark Bay in Western Australia.

**FIGURE 2:**
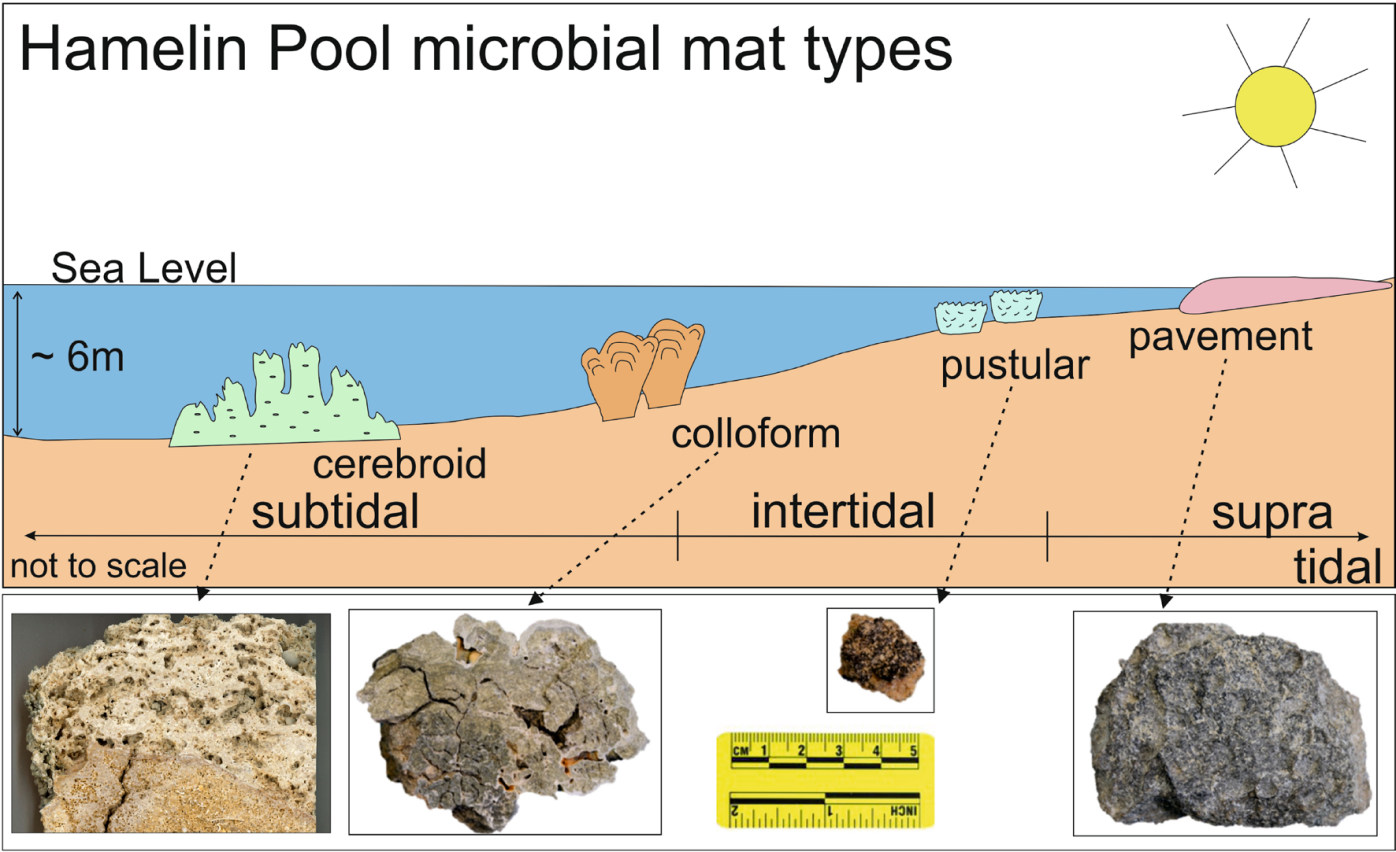
Schematic and simplified stromatolitic mat types of the hypersaline Hamelin Pool lagoon at Shark Bay related to water depth formation. Microbial pavement is characteristic of the supratidal environment. Pustular mat surfaces occur in the upper intertidal area; coarse laminated structures formed in colloform mat types are present in the lower intertidal to subtidal environment. Cerebroid structures are characteristic for the subtidal environment and represent the deepest lagoonal depositional setting. Mat types and related water‐depth formations are adapted from Jahnert and Collins (2012) and Vitek et al. (2023).

Hamelin Pool is located in the UNESCO World Heritage Site of Shark Bay in Western Australia (Figure 1), which is a ~1400 km^2^ lagoonal embayment with a ~135 km coastline populated and dominated by diverse flourishing microbial communities (e.g., Babilonia et al. 2018; Jahnert and Collins 2012; Reid et al. 2024). The lagoon is restricted to the open ocean in the north by the Faure Sill, building up a carbonate‐sand and seagrass bank. Environmental stress from hypersaline conditions, combined with temperature fluctuations and partial subaerial exposure, allows widespread microbial populations along the lagoon to persist without predatory pressure from macroalgae or eukaryotes (e.g., Reid et al. 2024; Suosaari, Reid, Playford, et al. 2016). The presence of actively forming up to 2000‐year‐old stromatolites at Shark Bay has been reported since the 1960s. Numerous studies have examined microbial diversity and stromatolite morphologies across different micro‐ and macro‐environments, shelf bathymetry, and water energy at Hamelin Pool (e.g., Jahnert and Collins 2012; Reid et al. 2024; Suosaari, Reid, Playford, et al. 2016; Suosaari et al. 2019). Microbial structures occur as poorly lithified laminated and non‐laminated sheets as well as lithified discrete build‐ups and pavements at Shark Bay (e.g., Jahnert and Collins 2012; Reid et al. 2024; Suosaari, Reid, Playford, et al. 2016; Vitek et al. 2023). Mat types of lithified stromatolite build‐ups can be further subdivided into (i) smooth, (ii) pustular, (iii) colloform, and (iv) cerebroid, which can be directly linked to formation at specific water depths and microbial community assemblages (Figure 2, e.g., Jahnert and Collins 2012; Vitek et al. 2023). We here follow the mat type description with related microbial consortia described by Jahnert and Collins (2012) and Vitek et al. (2023) for Shark Bay stromatolites: (i) Smooth mat types are finely laminated and predominantly occur in the lower intertidal zone, although the here sampled smooth stromatolite represents microbial pavement in the supratidal zone. Common microbial communities include *Chroococcus munutus* and *turgidus*. Microbial pavements are low‐relief, smooth lithified deposits with tabular or blocky surface morphology that are now exposed in the supratidal zone around Shark Bay due to sea‐level fall since the mid‐Holocene (Jahnert and Collins 2012; Martin et al. 2023). (ii) Pustular mat types show an irregular, clotted, blistered surface and reflect microbial build‐ups in the upper intertidal zone. Cyanobacteria found in this mat type are *Entophysalis granulosa, Gloeocapsa punctata*, and *Chroococcus* species such as *minutus, turgidus, microscopicus*, and *prescotti*. (iii) Coarse laminated fabrics formed in colloform mat types exhibit a hemispherical, globular morphology in intertidal and subtidal zones. This mat type is populated by *Entophysalis granulosa, Gloeocapsa punctata* and v*ibrio, Cyanosarcina thalassia, Aphanocapsa litoralis*, and the *Chroococcus* species *turgidus, prescotti*, and *ergovici*. (iv) Cerebroid structures contain irregular fenestrae fabrics with voids and reflect subtidal build‐ups in the hypersaline Shark Bay lagoon. Cyanobacteria consortia include *Gloeocapsa punctata, Aphanocapsa litoralis*, and the *Chroococcus* species *minutus, turgidius, prescotti, ergovici, giganteus*, and *turicensis*. Notably, stromatolites at Hamelin Pool in Shark Bay exhibit growth rates of < 1 to 50 cm per 1000 years (Chivas et al. 1990; Jahnert and Collins 2012). Most of these stromatolites have formed over time by various microbial communities under different environmental conditions and water depths (e.g., Jahnert and Collins 2012; Suosaari, Reid, Playford, et al. 2016; Suosaari et al. 2019, 2022). They represent a mixture of morphologies, internal fabrics, and depositional settings (Jahnert and Collins 2012; Suosaari, Reid, Playford, et al. 2016; Vitek et al. 2022, 2023).

## 3 Materials and Methods

### 3.1 Sampling Strategy and Description

All stromatolite samples from subtidal, intertidal, and supratidal environments at Hamelin Pool in Shark Bay, as well as ambient lagoon waters, were sampled and originally described by Martin et al. (2023). Sampling locations are shown in Figure 1. Stromatolitic carbonates are primarily composed of aragonite (> 90%) with minor calcite and quartz, as well as shell fragments (Figure S1; Martin et al. 2023). Sample powders from Martin et al. (2023) and additionally produced new powders of individual laminae/parts of stromatolite hand specimens, SB19‐1‐1 and SB19‐2‐1, were obtained with a hand‐held diamond‐coated microdrill. SB19‐1‐1 is a stromatolite from the intertidal zone with coarse laminated fabrics produced by colloform mat types (location 1; Figure S1). The upper part of the hand specimen (FUM6, FUM7) shows a light greenish color and can be described as a coarse, laminated fabric with fenestrae porosity, produced by a colloform mat type. The lower part (FUM8‐10) has a whitish‐beige color and a non‐laminated, more compact cerebroid structure, with fenestrae. This stromatolite sample most likely covers 300–400 years of growth, considering a growth rate of 0.3 mm/year (Chivas et al. 1990). Samples SB19‐1‐2x, SB19‐1‐4x (both colloform), and SB19‐1‐3b (pustular) are different stromatolite build‐ups from the exact sampling location (Figure S1, Martin et al. 2023). SB19‐2‐1 formed in a subtidal environment at location 2 and is a fenestrae‐rich, whitish‐beige, weakly laminated cerebroid microbial crust (FUM1‐3) that grows on a clastic sedimentary sand‐carbonate substrate with shell fragments (FUM5) (Figure S1). SB19‐2‐2x was also sampled from a stromatolite with cerebroid structure from locality 2 in the subtidal environment. Stromatolite sample SB19‐3‐1 from a supratidal environment at location 3 is a smooth mat interpreted as microbial pavement (Jahnert and Collins 2012; Martin et al. 2023), although smooth microbial mat types in Shark Bay are commonly found in intertidal environments (cf. Jahnert and Collins 2012; Vitek et al. 2023). Two lagoon waters and a shallow groundwater sample were sampled by Martin et al. (2023) in direct association with the stromatolite samples at all three localities. Temperature, pH, conductivity, dissolved oxygen, and oxidation–reduction potential (ORP) were directly measured at the sampling site, and all water samples were filtered through a 0.45 μm cellulose acetate filter and acidified with suprapure, concentrated HNO_3_ to a pH of ~2 (cf. Martin et al. 2023). Notably, both lagoon water samples have pH values of ~8.1, ORP values of ~200, and salinity values of 60–65 PSU, relative to the groundwater sample with a pH of 7.3, ORP of 93, and salinity of 3.6 PSU (Martin et al. 2023).

### 3.2 Analytical Methods

#### 3.2.1 δ^18^O_carb_ and δ^13^C_carb_ Values

Carbon (δ^13^C_carb_) and oxygen (δ^18^O_carb_) isotope compositions of the eight newly produced sample powders, as well as the carbonate standard material JDo‐1, were analyzed on a Thermo Delta V mass spectrometer equipped with a GASBENCH‐II preparation device following the approach previously described in (Martin et al. 2023). Briefly, ca. 20–50 μg of carbonate powder was weighed into a He‐filled 12 mL exetainer vial and dissolved in (H_2_O‐free) H_3_PO_4_ at 70°C. Subsequently, the CO_2_‐He gas mixture is transported to the GASBENCH using He as the carrier gas. Isotope compositions are given as δ^13^C_carb_ and δ^18^O_carb_ values. They are calibrated and reported relative to Vienna Pee Dee Belemnite (VPDB) using the international reference material NBS18, which is commonly used to calibrate stable isotope measurements. A total of 20 replicates of two in‐house CaCO_3_ standards are analysed in each run of 55 samples. An internal CaCO_3_ reference material (VICS; δ^13^C_carb_ = 1.45‰ VPDB, δ^18^O_carb_ = −5.44‰ VPDB) was measured alongside samples during the analytical run. Standard masses were selected to bracket the full range of sample mass. Following normalization for mass‐dependent isotope effects, standard reproducibility was < 0.1‰ for both the δ^18^O_carb_ and δ^13^C_carb_ values.

#### 3.2.2 Major and Trace Elements of Shark Bay Stromatolites and Water

Major and trace element data were obtained for stromatolitic carbonates and ambient water samples. Chemical digestion and analytical procedures differ between the two sample types and are described separately below. All acids used were of supra‐pure quality.

##### 3.2.2.1 Stromatolitic Carbonates

1. *Carbonate leaching*: Circa 200 mg of homogenous stromatolitic carbonate and substrate powder, and the carbonate reference material JDo‐1 were weighed in 15 mL centrifuge vials and washed with 10 mL milliQ water (MQ). The solutions were centrifuged, and the supernatants were discarded to remove the non‐carbonate fraction leachable with MQ water. Notably, the non‐quantitative measurement of the MQ‐leached fraction contains significant amounts of lithium, strontium, barium, and uranium, but no measurable REY concentrations. The remaining material was leached with 12 mL of 1 M supra‐pure acetic acid (HAc) to attack the carbonate fraction. Note that this fraction is mainly composed of stromatolitic carbonate, but may also include some carbonate allochems that may vary between the different stromatolite morphologies (cf. Vitek et al. 2022, 2023). The centrifuge vial was continuously shaken on a shaker for 1 h, and the solution was then centrifuged again. The solution was pipetted into Savillex beakers and immediately evaporated to dryness at 100°C. The evaporated carbonate fraction was then re‐dissolved in 10 mL of 3% HNO_3_. Aliquots of this solution were used for major and trace element analyses via Varian ICP‐OES and a ThermoFinnigan Element XR Sector Field ICP‐MS, respectively, as shown in Table S1.
2. *REY preconcentration and matrix separation method*: The remaining sample solutions were used for the REY preconcentration and matrix separation procedure, which was applied before spectrometric analysis to determine reliable REY data and avoid matrix effect interferences. The REY‐matrix separation protocol is based on Shabani et al. (1992) and has been routinely applied in its modified versions for different sample types, including river waters (e.g., Tepe and Bau 2014; Zocher et al. 2024), biogenic carbonates (e.g., Zhang et al. 2024) and plants (e.g., Zocher et al. 2021). The most important steps of the procedure are summarized below. Details can be found in the respective publications. The solutions were filtered through a 0.2 μm cellulose acetate filter, gravimetrically diluted to 1000 g with MQ, and acidified to a pH of ~2 with concentrated, supra‐pure HCl. This diluted sample solution was spiked with a Tm tracer to achieve 25 ng/kg. 50 mL of this solution was taken to monitor spike recovery during matrix separation and elution. The remaining solutions were passed through ion‐exchange columns (Sep‐Pak C18 cartridges) containing ethyl‐hexyl phosphates. The columns were washed with 10 mL of 0.01 M HCl to remove matrix elements and subsequently with 40 mL of 6 M HCl to eluate the REY from the columns. The eluates were evaporated and filled up to 10 g with 0.5 M HNO_3_. Measurements of REY were conducted with a PerkinElmer NexION 350x coupled with an APEX 2Q desolvating system. Spike recovery ranged from 97% to 107%, and measured REY concentrations were corrected accordingly (Table 1). Relative standard deviations of REY concentration measurements are below 1.3%, and blank concentrations for the REY series are well below 1 ng/g. REY concentrations of the JDo‐1 in this study are homogenously 44% ± 5% lower than published values (Table S2). This is expected given the acetic acid leaching procedure used here, compared with the complete digestion reported in the published values (cf. Table S2). The reliable REY data quality of our method is further corroborated by sub‐parallel REY_SN_ patterns between the published and our JDo‐1 data, indicating leaching of only the carbonate fraction and no significant REY fractionation during the applied laboratory procedure (Figure S2).

**TABLE 1:** Rey concentrations of stromatolites and waters of Shark Bay.

LOQ	Sample‐ID	Stromtolites																Waters		
		FUM6	FUM7a	SB19‐1‐2x	SB19‐1‐4x	FUM8	FUM9	FUM10	SB19‐1‐3b	SB19‐2‐2x	FUM1	FUM2	FUM3a	Fum 3b	FUM5	SB19‐3‐1	FUM‐SBS	SB19‐1 SW	SB19‐2 SW	SB19‐3 GW
	Morphology	Colloform	Colloform	Colloform	Colloform	Cerebroid	Cerebroid	Cerebroid	Pustular	Cerebroid	Cerebroid	Cerebroid	Cerebroid	Cerebroid	Substrate	Smooth	Beach sand	Lagoon water		Groundwater
	Setting	Intertidal								Subtidal						Supratidal		Intertidal	Subtidal	Supratidal
	Specimen	SB19‐1‐1	SB19‐1‐1	SB19‐1‐2x	SB19‐1‐4x	SB19‐1‐1	SB19‐1‐1	SB19‐1‐1	SB19‐1‐3b	SB19‐2‐2x	SB19‐2‐1	SB19‐2‐1	SB19‐2‐1	SB19‐2‐1	SB19‐2‐1	SB19‐3‐1				
ppb		ppm								ppm								ppt		
	Tm yield %	97	100	100	100	107	103	102	106	101	100	100	100	98	107	101				
1.05	Y	0.0207	0.0226	0.04	0.0376	0.187	0.191	0.178	0.105	0.148	0.0802	0.0841	0.0805	0.0709	1.11	0.121	0.297	2.004	1.688	0.127
1.20	La	0.0113	0.0152	0.0176	0.0143	0.237	0.244	0.228	0.085	0.142	0.059	0.0513	0.0612	0.0477	1.01	0.162	0.268	4.452	1.2545	0.0905
0.98	Ce	0.0262	0.0359	0.0393	0.0324	0.62	0.634	0.594	0.216	0.356	0.154	0.133	0.156	0.131	2.23	0.375	0.593	4.51	2.7375	0.151
0.16	Pr	0.00306	0.00413	0.00505	0.00392	0.0703	0.0721	0.0676	0.0249	0.0398	0.017	0.0149	0.0176	0.0135	0.288	0.0432	0.0656	0.785	0.3155	0.0175
0.49	Nd	0.0137	0.0185	0.0244	0.0184	0.284	0.298	0.283	0.103	0.162	0.0714	0.0621	0.0733	0.0568	1.14	0.181	0.252	2.58	1.22	0.068
< 2	Sm	0.00314	0.00424	0.00587	0.00472	0.0582	0.0618	0.0583	0.0224	0.0349	0.0156	0.0139	0.016	0.0125	0.244	0.0369	0.0532	0.511	0.2435	0.0145
0.03	Eu	0.000809	0.00101	0.00147	0.00125	0.0126	0.0134	0.0128	0.00502	0.00779	0.0035	0.00325	0.00359	0.00279	0.0546	0.00806	0.0145	0.095	0.0575	0.00297
0.18	Gd	0.00411	0.00499	0.00782	0.00666	0.0553	0.0587	0.0565	0.0229	0.0353	0.0163	0.0152	0.0172	0.0138	0.25	0.0366		0.414	0.25	0.015
0.03	Tb	0.000592	0.000643	0.00108	0.000975	0.00728	0.00771	0.00709	0.00308	0.0047	0.0022	0.00218	0.00232	0.00185	0.0348	0.00449	0.00843	0.083	0.038	
< 2	Dy	0.00368	0.00414	0.00744	0.00647	0.0393	0.0418	0.0393	0.0179	0.0265	0.0133	0.0131	0.0139	0.012	0.2	0.0242	0.0435	0.296	0.223	0.0135
0.04	Ho	0.000777	0.00087	0.00161	0.00147	0.0072	0.00763	0.0074	0.00347	0.00493	0.0027	0.00276	0.0028	0.00241	0.0383	0.0045	0.00968	0.085	0.05	0.004
< 2	Er	0.00247	0.0026	0.005	0.00433	0.0197	0.0206	0.0197	0.00975	0.0136	0.00803	0.00839	0.00836	0.00707	0.107	0.0116	0.0243	0.193	0.148	
Spike	Tm	Spike	Spike	Spike	Spike	Spike	Spike	Spike	Spike	Spike	Spike	Spike	Spike	Spike	Spike	Spike	0.00546	IS	IS	IS
< 1	Yb	0.0019	0.00202	0.00398	0.00346	0.0143	0.0149	0.0142	0.00732	0.00992	0.0067	0.00734	0.00681	0.0061	0.0811	0.00795		0.18	0.147	0.0125
0.01	Lu	0.000294	0.000313	0.000624	0.000492	0.00195	0.00201	0.00193	0.00105	0.00134	0.00104	0.00112	0.000991	0.000851	0.0111	0.00108	0.00468	0.046	0.0245	
	Y/Ho	26.6	26.0	24.8	25.6	26.0	25.0	24.1	30.3	30.0	29.7	30.5	28.8	29.4	29.0	26.9	30.7	23.6	33.9	33.2
	Ce_SN_/Ce*_SN_	1.14	1.16	1.16	1.18	1.03	1.06	1.07	1.04	1.06	1.11	1.08	1.07	1.19	0.89	1.06	1.00	0.70	1.45	1.40
	Gd_SN_/Gd*_SN_	1.19	1.38	1.40	1.26	1.14	1.15	1.22	1.19	1.17	1.23	1.15	1.22	1.34	1.14	1.22		0.59	1.06	0.90
	La_SN_/La*_SN_	1.28	1.28	1.67	1.52	0.87	0.92	0.95	0.93	0.94	0.99	0.96	0.97	1.01	0.86	1.06	0.95	1.58	2.78	3.27
	Eu_SN_/Pr_SN_	2.16	2.00	2.38	2.61	1.47	1.52	1.55	1.65	1.60	1.68	1.78	1.67	1.69	1.55	1.53	1.80	0.99	1.49	
	Eu_SN_/Yb_SN_	1.11	1.31	0.96	0.94	2.30	2.35	2.35	1.79	2.05	1.36	1.16	1.38	1.19	1.76	2.65		1.38	1.02	

Abbreviation: LOQ, limit of quantification.

##### 3.2.2.2 Beach Sand

200 mg of beach sand (FUM‐SBS, location 3) from Hamelin Pool was weighed into a Savillex beaker and fully digested to estimate elemental concentrations of the most likely source of detrital aluminosilicate contamination. This sample was digested in 10 mL of a concentrated HNO_3_‐HCl–HF mixture (3:1:1) for 1 week at 140°C. Afterward, the solution was evaporated and re‐dissolved twice with 6 M HCl, then immediately evaporated. The dried material was re‐dissolved in 3% HNO_3_ for ICP‐MS and ICP‐OES measurements.

##### 3.2.2.3 Water Samples

Two lagoon water samples (SB19‐1SW, ‐2SW) and one groundwater sample from a seep at a few meters depth within the lagoon (SB19‐3GW) were sampled in direct association with the stromatolites at Hamelin Pool (Figure 1). The water samples were immediately filtered through a 0.45 μm cellulose acetate filter (see Martin et al. 2023 for more information). The filtered solution was acidified with concentrated supra‐pure HNO_3_ to a pH of ca. 2 for analysis of REY using a ThermoFisher iCAP‐TQ ICP‐MS/MS equipped with an Elemental Scientific Apex 2Q desolvating nebulizer. The tandem ICP‐MS was coupled to an Elemental Scientific seaFAST SP2 system for on‐line REY preconcentration and matrix removal. Details of the analytical procedure have been described previously (Kraemer et al. 2024). Data quality for REY was ensured through repeated analyses of the water standards SLRS‐6 (*n* = 3) and NASS‐7 (*n* = 3) (Table S2). The REY data for the water CRMs agree with published values, and the REY_SN_ patterns for both newly analyzed and published values overlap accordingly (Figure S2).

### 3.3 REY Anomaly and Partition Coefficient Calculation

REY concentrations are normalized to the Post‐Archean Australian Shale (PAAS; Taylor and McLennan 1985), the most commonly used shale composite in aqueous geochemistry, to compare the REY fractionation directly within waters and their chemical precipitates relative to the weathered and eroded clastic influx from the uppermost continental crust. The following formulas were used to quantify anomalous REY behavior:

(1) $${\mathrm{La}}_{\mathrm{SN}}/{\mathrm{La}}_{\mathrm{SN}}*={\mathrm{La}}_{\mathrm{SN}}/\left(3{\mathrm{Pr}}_{\mathrm{SN}}\;—2{\mathrm{Nd}}_{\mathrm{SN}}\right)$$

(2) $${\mathrm{Ce}}_{\mathrm{SN}}/{\mathrm{Ce}}_{\mathrm{SN}}*={\mathrm{Ce}}_{\mathrm{SN}}/\left(2{\mathrm{Pr}}_{\mathrm{SN}}—{\mathrm{Nd}}_{\mathrm{SN}}\right)$$

(3) $${\mathrm{Gd}}_{\mathrm{SN}}/{\mathrm{Gd}}_{\mathrm{SN}}*={\mathrm{Gd}}_{\mathrm{SN}}/\left(2{\mathrm{Tb}}_{\mathrm{SN}}—{\mathrm{Dy}}_{\mathrm{SN}}\right)$$

Enrichment of middle REY_SN_ (MREY) relative to heavy REY_SN_ (HREY) and light REY_SN_ (LREY) and Y_SN_ anomalies was quantified using Eu_SN_ /Yb_SN_ and Eu_SN_/Pr_SN_ ratios as well as Y/Ho ratios, respectively. REY concentrations are also normalized to average sedimentary organic matter from different aqueous environments (Freslon et al. 2014).

Partition coefficients (Kd) determining fractionation of REY between stromatolitic carbonate and ambient waters at each location were calculated relative to Ca based on Webb and Kamber (2000), following the equation:

(4) $${\text{KdREY}}_{\left(\text{stromatolite}/\text{fluid}\right)}=\left({\mathrm{REY}}_{\text{stromatolite}}/{\mathrm{REY}}_{\text{water}}\right)*\left({\mathrm{Ca}}_{\text{water}}/{\mathrm{Ca}}_{\text{stromatolite}}\right)$$

Partition coefficients for the individual REY and associated calcium concentrations for the stromatolites and water samples are given in Table S3. Calcium data of the water samples are taken from Martin et al. (2023) and are 604 mg/L (SB19‐1SW) and 570 mg/L (SB19‐2SW) for the lagoon water samples, and 102 mg/L for the lagoon water sample with groundwater inflow (SB19‐3GW), respectively.

## 4 Results

### 4.1 δ^18^O_carb_ and δ^13^C_carb_ Values

**FIGURE 3:**
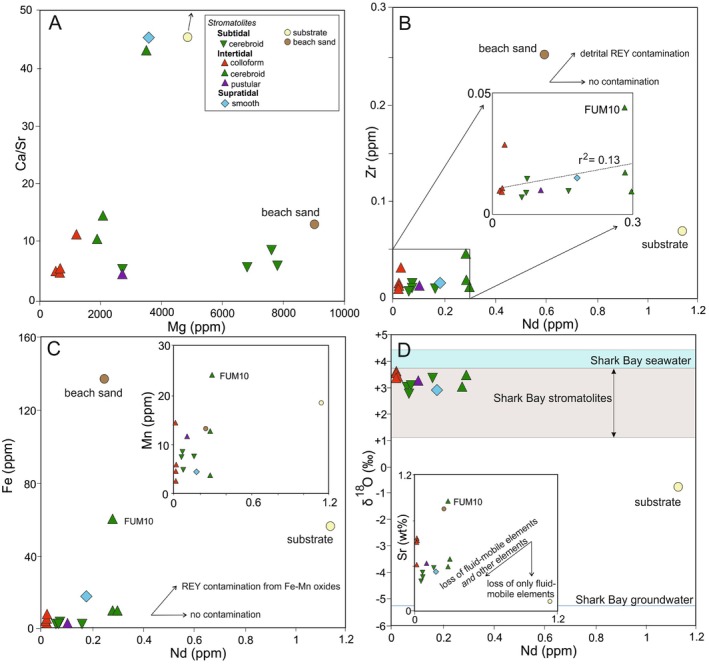
Plot of Ca/Sr ratios versus magnesium concentrations (A), and neodymium as representative of the REY series relative to zirconium concentrations (B), iron and manganese concentrations (C), and δ^18^O_carb_ values and strontium concentrations (D). (A) Ca/Sr ratios of most stromatolites of the intertidal and subtidal environments cluster around ~10 ± 5 at variable Mg concentrations that are different from the smooth microbial pavement of the supratidal environment, beach sand, and substrate. (B) The lack of correlation between immobile element concentrations, such as zirconium, and REY indicates negligible detrital aluminosilicate contamination. (C) The lack of iron and manganese (inset) with REY concentrations also provides evidence for the insignificant impact of Fe‐Mn oxides on the REY compositions of the stromatolitic carbonates. (D) The lack of correlation between REY and fluid‐mobile indicators, such as strontium concentrations or δ^18^O_carb_ values, indicates negligible post‐depositional fluid‐rock alteration.

δ^13^C_carb_ and δ^18^O_carb_ values of eight stromatolitic carbonates and the reference material JDo‐1 are reported in Table S1 in addition to data that are published in Martin et al. (2023). Newly analyzed samples include four colloform and three cerebroid stromatolite samples from the intertidal environment, as well as four cerebroid stromatolites and the substrate from the subtidal environment. δ^13^C_carb_ values from the intertidal stromatolite samples (SB19‐1) range from 5.04 ± 0.02 to 6.13‰ ± 0.02‰ VPDB and in the subtidal stromatolites (SB19‐2) from 4.76 ± 0.01 to 4.96‰ ± 0.03‰ VPDB. These δ^13^C_carb_ values are significantly higher than those of the ambient clastic substrate, representing the subsurface geology (δ^13^C_carb_ = −1.04‰ ± 0.05‰ VPDB; Table S1). δ^18^O_carb_ values range from 3.00 ± 0.02 to 3.54‰ ± 0.03‰ VPDB for the intertidal stromatolites and overlap with the δ^18^O values (2.80 ± 0.04 to 3.09‰ ± 0.01‰ VPDB) of the subtidal stromatolites. The substrate again differs strongly from the stromatolites and shows a negative δ^18^O_carb_ value of −0.79‰ ± 0.04‰ VPDB. The δ^13^C_carb_ and δ^18^O_carb_ values of 5.3‰ and 2.9‰ VPDB, respectively, from the supratidal sample are reported by Martin et al. (2023). Lagoon water samples SB19‐1SW and SB19‐2SW have δ^18^O values of 3.8‰ and 4.3‰ VSMOW, respectively, while the groundwater sample SB19‐3GW shows a δ^18^O value of −5.3‰ VSMOW (Martin et al. 2023). Assuming a water temperature of 22°C, carbonate precipitated from these waters would yield aragonite δ^18^O ranging from 3.29‰ to 2.79‰ VPDB values (calculated using Kim et al. 2007) that directly overlap with those of the stromatolites (Figure 3). Notably, all δ^18^O_carb_ and δ^13^C_carb_ values of the Shark Bay stromatolites reported here are in the range of previously reported δ^18^O_carb_ and δ^13^C_carb_ values of Chivas et al. (1990), Jahnert and Collins (2012), and Martin et al. (2023).

### 4.2 Major Elements

Major and trace element data of acetic acid leachates of the stromatolitic carbonates and the substrate from the subtidal, intertidal, and supratidal environments of Shark Bay, as well as the fully digested beach sand from location 3 at Hamelin Pool, are given in Table S1. Stromatolites show calcium, magnesium, and strontium concentrations in the wt% range. Calcium concentrations range from 1.68 to 15.7 wt% (except FUM10 with 41.2 wt%). The stromatolite sample with smooth fabric from the supratidal environment shows the highest calcium concentration (15.7 wt%), followed by cerebroid stromatolites from the intertidal setting with somewhat higher concentrations (4.64–5.59 wt%; exception FUM10). Colloform and pustular stromatolites collected from the intertidal environment and stromatolites with cerebroid textures from the subtidal environment have lower calcium concentrations between 1.68 and 3.1 wt% calcium (one exception, SB19_1‐2x, with 6.72 wt%). The substrate (18.3 wt% calcium) and beach sand (11.7 wt% calcium) generally have higher calcium concentrations relative to the stromatolites. Magnesium concentrations in the stromatolites range from 0.06 to 0.78 wt% magnesium, with the lowest magnesium concentrations found in colloform textures of the intertidal setting (< 0.12 wt% magnesium) (Figure 3). The magnesium concentration of the substrate (0.48 wt% Mg) falls into the range of the stromatolites. In contrast, the magnesium concentration of the beach sand (0.9 wt% magnesium) is slightly elevated relative to stromatolites. Strontium concentrations range from 0.27 to 0.96 wt% in the stromatolites relative to 0.09 wt% strontium in the substrate and 0.9 wt% strontium in the beach sand. Ca/Sr ratios are highest in the smooth stromatolite from the supratidal environment (Ca/Sr = 45.4, Figure 3). Stromatolites with cerebroid textures from the intertidal environment have Ca/Sr ratios above 10. In contrast, all other stromatolite samples have Ca/Sr ratios below 10 (one exception, SB19_1‐2x with Ca/Sr = 11). The beach sand (13) and also the substrate (209) have elevated Ca/Sr ratios relative to most stromatolites.

The iron and manganese concentrations in the stromatolites are in the lower ppm range. Iron concentrations in all stromatolites range from 0.643 to 17.6 ppm (exception FUM10 with 58.6 ppm iron) and are lower than those of the substrate (55.8 ppm iron) and beach sand (136 ppm iron) (Figure 3). Manganese concentrations are also in the lower ppm range and unsystematically distributed between stromatolites (2.35 and 24 ppm manganese), the substrate (18.5 ppm manganese), and beach sand (13.2 ppm manganese) (Figure 3). Aluminum concentrations in the stromatolites are typically below 20 ppm, with the exceptions of the smooth stromatolite from the supratidal environment (44.6 ppm aluminum) and one cerebroid stromatolite from the intertidal environment FUM10 (100 ppm aluminum). The substrate (249 ppm) and beach sand (147 ppm) show higher aluminum concentrations than the stromatolites. Notably, phosphorus concentrations were consistently below the detection limit of 56 ppm.

### 4.3 Trace Elements, REY Anomalies, and REY Partition Coefficients Between Waters and Carbonates

Acetic acid leachates of stromatolitic carbonates show very low immobile trace element concentrations. For instance, zirconium (7–43.6 ppb), thorium (below the limit of quantification (LOQ) of 0.7–12 ppb, with the exception FUM10 with 171 ppb thorium), and hafnium (below LOQ of 6 ppb) (Table S1; Figure 3) are all in the lower ppb range consistent with very low aluminum concentrations. The acetic acid leachate of the substrate (14.9 ppb zirconium, 12.7 ppb thorium, 18.6 ppb hafnium) is only slightly enriched in these immobile elements relative to the stromatolites. At the same time, the fully digested beach sand shows, as expected for full digestions, orders of magnitude higher immobile element concentrations (594 ppb zirconium, 140 ppb thorium, 10.4 ppb hafnium). Rare earth element and yttrium concentrations in stromatolitic carbonates obtained by acetic acid leaching followed by REY preconcentration and matrix separation are in the lower ppb range (Table 1). The highest REY concentrations are reported from the cerebroid stromatolites of the intertidal environment and the smooth stromatolite from the supratidal environment (Figure 3, Table 1). Intermediate REY concentration values are reported from the pustular stromatolite of the intertidal environment as well as from cerebroid stromatolites of the subtidal environment; lowest REY concentrations are found in intertidal stromatolites with colloform structures. The REY concentrations of the stromatolites are lower relative to the ambient beach sand and substantially lower than the REY concentrations of the substrate. Lagoon water at location 1 (SB19‐1SW) has somewhat higher REY concentrations relative to the lagoon water at location 2 (SB19‐2SW; Table 1). Both lagoon water samples have much higher REY concentrations relative to the lagoon water‐groundwater mixture at location 3 (SB19‐3GW).

**FIGURE 4:**
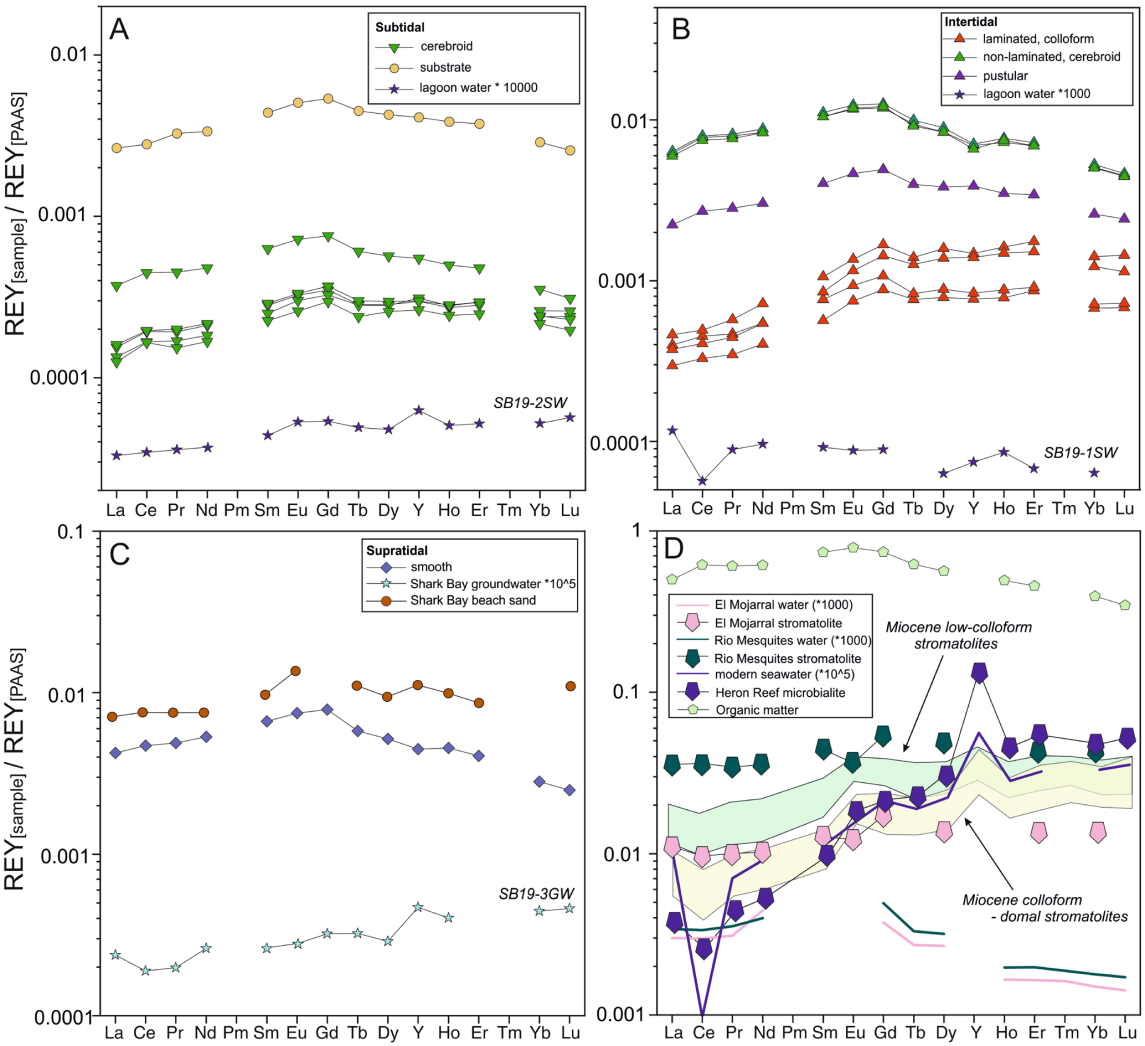
Shale‐normalized REY patterns of stromatolites and ambient waters of subtidal (A), intertidal (B), and supratidal (C) environments at Shark Bay relative to REY signatures of modern seawater, organic matter, Mexican waters, and ambient stromatolites and Phanerozoic microbialites (D). REY_SN_ patterns of all environments and morphologies at Shark Bay are different from those of modern seawater. All stromatolites show MREY_SN_ enrichment relative to LREY_SN_ and (except colloform morphologies) also to the HREY_SN_. See text for discussion. Data in (D) are taken from Webb and Kamber (2000), Douville et al. (2002), Johannesson et al. (2014), Freslon et al. (2014), and Viehmann et al. (2023).

La_SN_/La_SN_* and Gd_SN_/Gd_SN_* ratios of stromatolites are in the range of 0.87 to 1.67 and 1.14 to 1.40, respectively. Negative Ce_SN_ anomalies are not observed, but some carbonates show slightly positive Ce_SN_/Ce_SN_* ratios, ranging between 1.03 and 1.19 (Table 1). Y/Ho ratios in the range of 24.1 to 30.5 are slightly sub‐ to super‐chondritic. All stromatolites show MREY_SN_ relative to LREY_SN_ enrichments (Eu_SN_/Pr_SN_ between 1.47 and 2.61). Sometimes MREY_SN_ are also enriched relative to HREY_SN_ (Eu_SN_/Yb_SN_ = 0.94–2.65) (Figure 4). Water samples from Shark Bay show positive La_SN_ anomalies (La_SN_/La_SN_* = 1.58–3.27) but lack pronounced positive Gd_SN_ anomalies (Gd_SN_/Gd_SN_* = 0.59–1.06). Y/Ho ratios range from sub‐to slightly super‐chondritic (Y/Ho = 23.6–31.6).

**FIGURE 5:**
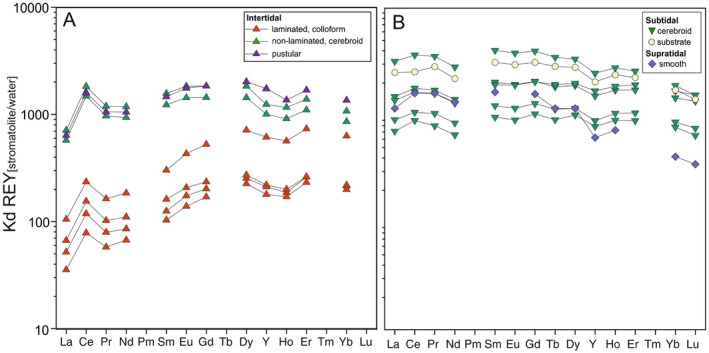
Kd_(stromatolite‐fluid)_ for the REY series of the intertidal (A) and subtidal and supratidal environments (B) at Shark Bay. (A) Cerebroid and pustular structures show higher and more consistent Kd values relative to colloform stromatolite structures. Colloform structures have a pronounced lower Kd for LREY relative to HREY. (B) Cerebroid structures have the most constant Kd value for individual REY throughout the REY series of all the sampled environments and stromatolite structures. The smooth structure, i.e., the microbial pavement, exhibits a lower Kd for HREY than other structures. Note that redox‐dependent Ce enrichments are not taken into account when interpreting Kd values.

Calcium‐normalized REY partition coefficients (cf. Webb and Kamber 2000) between stromatolitic carbonate and ambient waters at Shark Bay are in the range of Kd_(stromatolite/fluid)_ = 35 and 4034 (Table S3). The lowest overall Kd_(stromatolite/fluid)_ values are observed in intertidal environments (Figure 5; Table S3). Supratidal and, in particular, subtidal environments show the highest overall Kd_(stromatolite/fluid)_ values at Shark Bay. Coarse laminated fabrics of colloform mat types (Yb_Kd_/Pr_Kd_ > 2.1) and pustular structures (Yb_Kd_/Pr_Kd_ = 1.3) typically show an enrichment in HREY relative to MREY and LREY; the stromatolite with smooth morphology shows a depletion of HREY (Yb_Kd_/Pr_Kd_ > 0.3) relative to LREY. Cerebroid morphologies generally show the most uniform Kd_(stromatolite/fluid)_ values (Yb_Kd_/Pr_Kd_ between 0.8 and 1.1) along the REY series.

## 5 Discussion

### 5.1 Evaluation of Potential Contamination and Post‐Depositional Alteration of the REY Signatures in Hamelin Pool Stromatolites of Shark Bay

The prerequisite to use chemical sediments as geochemical archives to reconstruct paleo‐environments and their ambient physico‐chemical conditions strongly relies on the sample purity of the chemical sediment. If contamination by a detrital component is negligible and the authigenic component was not modified by post‐depositional alteration, the geochemical composition of the sediment may directly mirror the pristine chemical composition of the ancient fluid from which it precipitated. However, contamination by different terrigenous and secondary mineral phases that may have been potentially dissolved or leached by laboratory digestion and/or post‐depositional alteration processes that resulted from fluid–rock interactions during diagenesis or metamorphic overprints may have significantly masked the pristine geochemical compositions of the sediments (e.g., Webb and Kamber 2000; Schier et al. 2021). In the following, the purity of the stromatolitic carbonates from Shark Bay and potential processes that may have altered the pristine REY signatures in the carbonates are discussed in detail.

#### 5.1.1 Mineral contamination

Shark Bay carbonates show REY concentrations in the ppb range that are lower than those in ambient non‐stromatolitic material, such as beach sand or the substrate. These concentrations are also considerably lower than the REY concentrations of terrigenous detrital aluminosilicates, phosphates, and Fe‐Mn oxides that typically contain orders of magnitude higher REY concentrations (e.g., Schier et al. 2021). The dissolution of even trace amounts of these potential contaminants during stromatolitic carbonate digestion may significantly alter and mask the pristine REY signature of the carbonates. It is expected that if contamination of one or more of these mineral phases affected the REY composition of the stromatolitic carbonates, then the REY would show a positive correlation with respective element concentrations representing the individual mineral phases. Thus, a concurrent REY enrichment with concentrations of immobile elements such as aluminum is expected for detrital aluminosilicates, with iron and manganese for Fe‐Mn oxides, and/or with phosphorus for phosphates. Weak carbonate leaching of the stromatolitic carbonate powders with 1 M HAc in this study already prevented significant partial or complete dissolution of these mineral phases. Dissolution of these mineral phases is expected to be more severe under leaching with HCl or HNO_3_ digestion techniques (Schier et al. 2021). However, leaching of carbonate allochems that are present in variable amounts in the sampled stromatolite morphologies relative to micrite (cf. Vitek et al. 2022, 2023) cannot be entirely avoided with mild HAc leaching. The first evidence that carbonate allochems do not have a severe impact on stromatolite geochemistry in Shark Bay comes from δ^18^O_carb_ and δ^13^C_carb_ values. The stromatolite values differ markedly from those of the ambient substrate, which contains detrital carbonate grains and shell fragments. It is expected that if carbonate allochems derived from the ambient substrate, i.e., the most likely contaminant in this area, are dominating between 20% and 90% of the stromatolite fabrics (Vitek et al. 2022, 2023), then this contribution should have a significant impact on the respective δ^18^O_carb_ and δ^13^C_carb_ values in the stromatolites. Thus, these values should be much more heterogeneously distributed throughout the stromatolite morphologies based on the relative abundance of allochems in each specific stromatolite morphology type. This is not observed (Figure 3D, Table S1). Still, the comparison of REY with geochemically very different proxies, such as δ^18^O_carb_ and δ^13^C_carb_ values, is somewhat invalid and leaves minor uncertainties that cannot entirely be ruled out. Phosphorus concentrations below the detection limit within the stromatolitic carbonates suggest negligible REY contamination from the dissolution of secondary or terrigenous phosphates. Carbonate leachates also show very low immobile element concentrations (< 44.6 ppm aluminum and < 43 ppb zirconium) that are lower than in complete digestion of the pure limestone CRM JLs‐1 (> 50 ppm aluminum and ~300 ppb zirconium) or acetic acid leachates of the dolomite CRM JDo‐1 (13.7 ppm aluminum and 44 ppb zirconium; this study). Neodymium concentrations in stromatolitic carbonate leachates are chosen as representative of the REY series and show insignificant correlations with zirconium (Figure 3, *n* = 13, *p* = 0.21 at a significance level of 0.01) or aluminum (*n* = 13, *p* = 0.54 at a significance level of 0.01, not shown). Interestingly, the beach sand and substrate at Shark Bay also show comparably low immobile element concentrations, which is most likely related to individual sample mineralogy containing mostly carbonates, shells, and quartz (Figure 3 and Figure S1) that naturally contain low REY concentrations. In contrast, shale composites such as PAAS exhibit orders‐of‐magnitude higher immobile element concentrations (ca. 8 wt% aluminum and > 200 ppm zirconium; Taylor and McLennan 1985). The combination of very low immobile element concentrations with the lack of correlation of REY with immobile element concentrations strongly supports the absence of significant REY contamination by terrigenous detrital aluminosilicates in Shark Bay stromatolites. Similarly, very low iron and manganese concentrations within the ppm level range of the stromatolitic carbonates in combination with the lack of correlations between REY with iron (*n* = 13, *p* = 0.38 at a significance level of 0.01) or manganese (*n* = 13, *p* = 0.15 at a significance level of 0.01) concentrations (Figure 3), indicate that REY contamination in the stromatolites via dissolution of secondary Fe‐Mn oxides was negligible.

#### 5.1.2 Post‐depositional fluid‐rock alteration

REY in carbonates and other chemical sediments are very robust and immobile during fluid–rock interactions. Negligible REY mobility is even reported in Precambrian rock successions that experienced weak to severe metamorphic overprints (e.g., Van Kranendonk et al. 2002; Viehmann et al. 2020). Alteration of REY patterns by hydrothermal or metamorphic fluids requires water‐rock ratios in excess of 10^2^–10^3^ or severe metasomatic overprints to have a significant impact on the pristine REY composition (e.g., Banner and Hanson 1990). This is also true for REY signatures in carbonates during diagenetic alteration processes due to high partition coefficients between REY in carbonates and seawater above 100 (Zhong and Mucci 1995; Webb and Kamber 2000). Generally, low REY concentrations in diagenetic fluids require water‐rock ratios of > 104 to effectively alter REY patterns (Banner and Hanson 1990). Additionally, while fluid‐mobile elements such as strontium and oxygen, and their isotopes, can be significantly affected and redistributed during burial dolomitization, alteration of REY signatures is uncommon (Banner and Hanson 1988). This is supported by studies by Hood et al. (2018) and Liu et al. (2019), which show that REY signatures in carbonates are not strongly affected by meteoric or marine burial diagenesis despite mineralogical transformations. Stromatolitic carbonates of Shark Bay consist of > 90% primary aragonite (Martin et al. 2023), corroborated by comparably high strontium concentrations in the wt% range and Ca/Sr ratios < 45.4 (Figure 3). In contrast, the substrate almost entirely consists of calcite (Martin et al. 2023). It shows higher calcium concentrations at lower strontium concentrations and an elevated Ca/Sr ratio of 210 relative to stromatolites (Figure 3). Notably, no significant correlation between aragonite content of the overall stromatolite mineralogy (expressed as Ca/Sr ratios) and REY concentrations exists (*n* = 13, *p* = 0.52 at a significance level of 0.01; not shown). If post‐depositional alteration processes or mineralogical modifications of the original aragonite would have affected the REY composition in the stromatolitic carbonates, one would expect a decrease of fluid mobile element concentrations or their isotopic compositions together with REY. However, REY show no correlation with δ^18^O values of the stromatolites (*n* = 13, *p* = 0.43 at a significance level of 0.01; Figure 3) nor with strontium concentrations (*n* = 13, *p* = 0.59 at a significance level of 0.01; Figure 3D inset) that are especially prone to post‐depositional modifications if fluid–rock interactions occurred in the sample set. The combination of low correlations between REY and fluid‐mobile elements, with no prominent macroscopic or microscopic alteration features (Figure S1), indicates negligible REY remobilization during potential post‐depositional alteration processes.

Stromatolite sample FUM10 from the intertidal zone, however, has elevated immobile element concentrations of iron, manganese, and strontium. The reason for this remains speculative and cannot be resolved here, so this sample is removed from further discussion. All other stromatolite samples from Shark Bay are considered to have preserved their original REY composition and to be reliable archives for investigation of REY behavior in sub‐recent stromatolitic carbonates of the hypersaline Shark Bay lagoon.

### 5.2 Morphology‐Dependent REY Signatures in Stromatolites From Shark Bay

#### 5.2.1 REY Distributions in Shark Bay Lagoon Waters

The REY_SN_ patterns of subtidal, intertidal, and supratidal lagoon waters, as well as the groundwater ambient to the sampled stromatolites, are heterogeneous. They exhibit a smaller development of seawater‐like features (Figure 4, Table 1): The lagoon water sample from the subtidal environment shows small HREY_SN_ over LREY_SN_ enrichment and a slight positive Y_SN_ anomaly. The intertidal lagoon water sample shows non‐seawater‐like HREY_SN_ over LREY_SN_ depletion, but has a positive La_SN_ and a negative Ce_SN_ anomaly similar to modern seawater. The groundwater sample from the supratidal environment is most identical to the “typical” open‐ocean seawater‐like distribution, with an HREY_SN_ over LREY_SN_ enrichment and positive La_SN_ and Y_SN_ anomalies. This may reflect comparable REY speciation and fractionation behavior during sorption processes between REY solution complexes and surfaces, as observed in other aqueous systems with dominant mono‐ and dicarbonate REY complexation. Negative Ce_SN_ anomalies are commonly observed in sufficiently oxygenated fresh waters, such as in several worldwide rivers (e.g., Merschel, Bau, Schmidt, et al. 2017; Zocher et al. 2024) and groundwaters (Johannesson et al. 1997), but are absent in the groundwater sample from Shark Bay. Thus, the groundwater from the Tamala Limestone aquifer discharged into the Shark Bay lagoon must have been somewhat reducing and not been in exchange with the atmosphere. This is corroborated by a lower ORP value in groundwater compared with the other lagoon waters (cf. Martin et al. 2023).

The reason(s) for non‐seawater‐like REY_SN_ distributions in the hypersaline lagoon waters, however, remain speculative and may be a result of one or more combined processes. Processes responsible for the distinct REY distributions in lagoon waters are discussed in the following:

- One option to explain the variability in Shark Bay REY water chemistry is based on the 0.45 μm filter size. Different amounts and types of lithic and organic colloids, which affect the dissolved fraction passing through 0.45 μm filters, may dominate this fraction with different geochemical compositions. This would be similar to observations in river waters in which the dissolved loads are dominated by organic or lithic nanoparticle REY chemistry (Tepe and Bau 2014; Merschel, Bau, and Dantas 2017).
- Another option is based on REY speciation in the dissolved load, which was observed by Johannesson et al. (2014). These authors reported non‐seawater‐like REY_SN_ patterns with the lack of negative Ce_SN_ anomalies of mildly brackish waters of the Cuatro Ciénegas Basin, which is a critical groundwater discharge zone (Figure 4D). The authors calculated more dominant speciation of REY sulfate complexation in these waters relative to > 95% of mono‐ and di‐carbonate REY complexation that is observed in modern seawater (Johannesson et al. 2014). Unfortunately, anion concentrations for the water samples from Shark Bay are not available in our sample set, so speciation modeling to test different REY speciation at Shark Bay cannot be performed here.
- Alternatively, uneven and seasonal fluctuations of groundwater discharges, rain/river waters, or variable influxes of benthic flows via porewaters circulating and leaching the subsurface geology in the Shark Bay lagoon may account for variable lagoon water chemistry and short‐term salinity variability.

#### 5.2.2 Unevenly Distributed REY Partition Coefficients Between Stromatolite Morphologies and Ambient Waters at Shark Bay

##### 5.2.2.1 Contrasting REY Distributions Between Stromatolites and Ambient Waters

Webb and Kamber (2000) observed sub‐parallel REY_SN_ distributions between Holocene Great Barrier Reef microbialites and open ocean seawater with an average Kd for the REY series between carbonate and seawater of 295.5 (Figure 4D). The authors concluded that pure microbial carbonates are prime archives to reflect the chemistry of the fluid from which the carbonate precipitated. In contrast, Johannesson et al. (2014) reported contrasting REY_SN_ signatures in microbialites and ambient waters in a high groundwater discharge environment in the Cuatro Ciénegas Basin. These microbialites show distinct HREY_SN_ enrichment in the carbonate relative to ambient waters. This difference constrains the applicability of REY in carbonates as a direct paleo‐environmental proxy for reconstructing microbial habitats (Figure 4D).

The REY_SN_ patterns of all Shark Bay stromatolitic carbonate leachates are different from those of open ocean seawater (Figure 4). Typical open ocean seawater‐like REY_SN_, featuring prominent heavy REY_SN_ (HREY_SN_) over lighter REY_SN_ (LREY_SN_) enrichment, along with strong positive anomalies in La_SN_, Gd_SN_, and Y_SN_, are either absent or only faintly developed in stromatolitic carbonates (Figure 4, Table 1). Redox‐dependent strong negative Ce_SN_ anomalies occur due to Ce^3+^ oxidation to Ce^4+^ on particle and oxide surfaces, resulting in a subsequent depletion in the ambient fluid (e.g., Bau and Koschinsky 2009). Thus, negative Ce_SN_ anomalies are common in modern open ocean seawater (Ce_SN_/Ce_SN_* = 0.1) but are absent in the Shark Bay stromatolitic carbonates. Cerebroid stromatolites sometimes even develop slightly positive Ce_SN_ anomalies (Ce_SN_/Ce_SN_* = 1.03–1.19). Instead, stromatolitic carbonates of different depositional settings and with different morphologies show pronounced middle REY_SN_ (MREY_SN_) enrichment relative to the LREY_SN_ and HREY_SN_. Only coarse fabrics produced by colloform mat types of the intertidal environment show somewhat different REY_SN_ signatures relative to the other carbonates. These differences include a stronger pronounced MREY_SN_ relative to LREY_SN_ enrichment and a relatively flat MREY_SN_ to HREY_SN_ distribution (Figure 4, Table 1).

Thus, stromatolites of the hypersaline Shark Bay lagoon show contrasting REY_SN_ patterns between carbonates and ambient waters. However, it appears that REY partitioning into carbonates at Shark Bay, as reflected by the Kd value, can be directly related to stromatolite textures and morphology (Figure 5):

- Colloform stromatolites from the intertidal environment have the lowest and most variable Kd values between stromatolite and ambient water (excluding Ce due to redox sensitivity; cf. Webb and Kamber 2000). They also show the most substantial HREY enrichment relative to the LREY (Kd of Yb_Kd_/Pr_Kd_ = 2.1–3.9, Table S3).
- The pustular stromatolite from the intertidal environment shows a higher Kd value and smaller HREY over LREY enrichment (Yb_Kd_/Pr_Kd_ = 1.3) relative to the coarse laminated fabrics from colloform stromatolite mat types.
- Cerebroid stromatolites, independent of their intertidal or subtidal depositional environments, have the highest Kd values for the REY series between stromatolitic carbonates and ambient waters. They also show the closest similarities within the REY series with typical Yb_Kd_/Pr_Kd_ ratios between 1.1 and 0.8 (except SB19‐2‐2x). The Kd values for the REY series of the cerebroid morphologies > 875 (Table S3) are much higher at Shark Bay in comparison to Heron Reef, with 295.5 (Webb and Kamber 2000).
- The smooth stromatolitic carbonate, i.e., the microbial pavement from the supratidal area, has a Kd value comparable to the cerebroid structures but shows a substantial HREY depletion relative to LREY (Yb_Kd_/Pr_Kd_ = 0.3).

##### 5.2.2.2 Potential Mechanisms for REY Variability Recorded in Shark Bay Stromatolites

**FIGURE 6:**
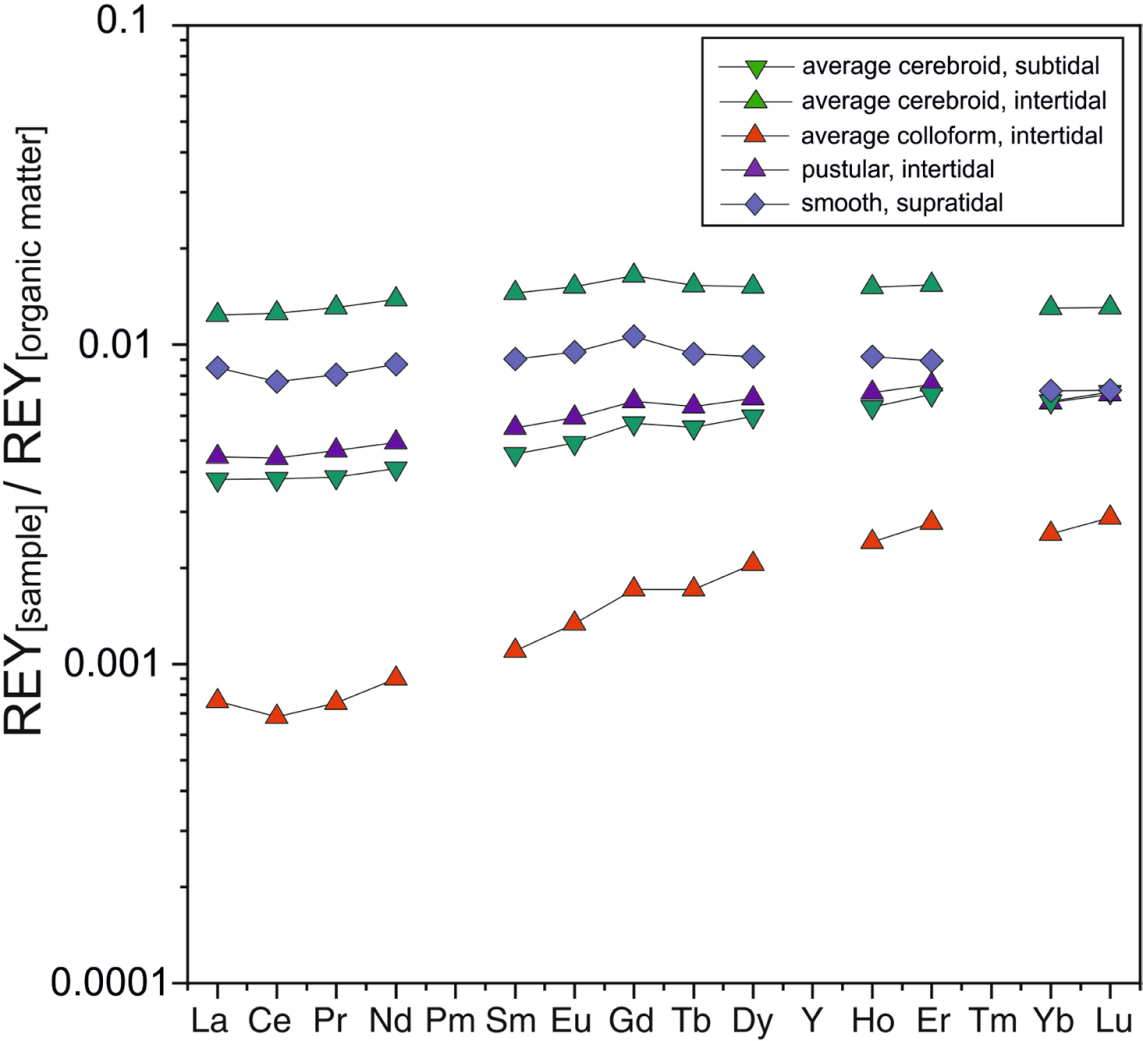
Organic matter‐normalized REY patterns of stromatolite morphologies at Shark Bay. Colloform structures show a HREY over MREY and LREY enrichment. In contrast, cerebroid, pustular, and smooth stromatolite morphologies show relatively flat organic matter‐normalized REY patterns, indicating that organic matter degradation in (semi)closed microbial mat systems had a significant impact on the REY geochemistry of the Shark Bay stromatolitic carbonates. Average organic matter data are from Freslon et al. (2014) and used to normalize against average REY concentrations across the different stromatolite morphologies of Shark Bay.

The variability and differences of the Kd values between the different stromatolite morphologies most likely reflect a combination of (i) different stromatolitic carbonate formation micro‐environments represented by the different morphologies, and (ii) that the modern Shark Bay water at the different sampling localities is not directly comparable with the ambient water from which the carbonate was formed. Indeed, Jahnert and Collins (2012) showed that short‐term sea‐level fluctuations in the Shark Bay lagoon affected stromatolite morphology, i.e., fluctuations in the lagoon water level resulted in stromatolitic textures that evolved from microbial pavement over cerebroid into colloform, smooth, and pustular textures within a single stromatolite dome. This morphological evolution was directly linked to water depths and micro‐environmental changes, spanning subtidal, intertidal, and supratidal carbonate‐forming environments. This also led to changes, adaptations, and diversification in the microbial communities that thrived at different water depths and are representative of different stromatolite morphologies at Shark Bay (e.g., Babilonia et al. 2018; Jahnert and Collins 2012; Reid et al. 2024; Suosaari, Reid, Playford, et al. 2016; Suosaari et al. 2019). Furthermore, the complexity and geo‐biological interactions within the stromatolite‐forming microbial mat systems, which are discussed in detail below, may play a crucial role for the REY geochemistry of carbonates that formed within the microbial mats:

1. Different REY speciation in specific microenvironments created by microbial metabolisms may result in distinct REY_SN_ patterns related to pH, alkalinity changes, and ion availability (Dupraz and Visscher 2005), thereby affecting LREY and HREY solution species. For instance, Johannesson et al. (2014) observed that not only mono‐ and di‐carbonate complexes affect the REY distribution in waters ambient to the Mexican stromatolites (Figure 4), but also dominant LREY sulfate complexes. In addition to REY carbonate and sulfate complexes, the availability of hydroxyl, phosphate, fluorite, and chloride complexes in low‐temperature aqueous systems affects dissolved REY distributions that are eventually incorporated into chemical sediments such as carbonates (e.g., Möller et al. 2021, and references therein). As mentioned above, anion data are unfortunately not available in our sample set for modeling REY speciation at Shark Bay.
2. REY are not considered to be bio‐essential elements to date, and the uptake and use of REY in microbial metabolisms are almost unknown. Thus, different microbial consortia with specific metabolisms present in different stromatolitic build‐ups (cf. Babilonia et al. 2018; Jahnert and Collins 2012) may account for differences in REY cycling and uptake.
3. Exopolymeric substances (EPS) produced by various bacteria within and on the microbial mat have been shown to concentrate trace metals such as Zn, Mn, Cu, and As (Sforna et al. 2016). EPS provides large quantities of surface functional groups that may also fractionate particle‐reactive species such as REY from ambient waters. Indeed, Takahashi et al. (2005) showed that the surface functional groups of bacterial cell walls exhibit an affinity for sorbing HREY relative to LREY. This observation led Johannesson et al. (2014) to conclude that these surface sites outcompete REY sulfate, bicarbonate, and chlorite complexes in the water, which, in turn, leads to a HREY_SN_ enrichment observed in Mexican stromatolites relative to ambient waters (Figure 4D). At Shark Bay, pustular, smooth, and cerebroid stromatolitic carbonates show a HREY_SN_ depletion relative to MREY_SN_ rather than enrichment, which makes this similar process unlikely. Only carbonates from coarse laminated colloform build‐ups show relatively flat MREY_SN_ to HREY_SN_ distribution and higher Kd values for the HREY relative to the LREY (Figure 5), which indicates that REY fractionation on EPS may play a more critical role for carbonate REY geochemistry in colloform structures relative to the other stromatolite morphologies at Shark Bay.
4. Organic matter has higher REY concentrations relative to the Shark Bay carbonates, a relatively flat REY_SN_ pattern with sometimes MREY_SN_ enrichment relative to the HREY_SN_ and LREY_SN_, and sometimes shows a positive Ce_SN_ anomaly independent of terrestrial or marine aqueous environment (Freslon et al. 2014; Figure 4D). Assuming organic matter degradation within microbial mats at Shark Bay (cf. Dupraz and Visscher 2005) and subsequent REY release within a closed microbial mat system may sufficiently impact the REY chemistry of the pore fluids and the stromatolitic carbonates that formed from this fluid. Breakdown of Mn oxides, with coincident Ce release from the oxide surfaces into ambient pore waters, has already been observed under anoxic conditions within the interior of microbial mats in Proterozoic *conophyton*‐type stromatolites of the ca. 1‐billion‐year‐old Paranoá Group in Brazil (Viehmann et al. 2019). A slight hint of a comparable process occurring in Shark Bay may be the positive Ce_SN_ anomaly in the stromatolitic carbonates, reflecting the breakdown of Mn‐Fe oxides or the degradation of organic matter, releasing REY, among other elements, into the microbial mat porewaters. The breakdown of Mn‐Fe oxides, however, is somewhat unlikely due to the lack of correlation between Mn (*n* = 13, *p* = 0.35 at a significance level of 0.01) and Fe concentrations (*n* = 13, *p* = 0.34 at a significance level of 0.01) with Ce/Ce* ratios. In contrast, organic matter such as EPS degradation within the semi‐closed microbial mat systems and the subsequent release of trace metals bound to this organic matter into the pore fluids are likely and have already been observed for Zn, Mn, Cu, or As, which eventually concentrate as metal‐enriched sulfides aligned at the stromatolite lamination (Sforna et al. 2016). Given the similarity of REY_SN_ patterns of organic matter to those of cerebroid, pustular, and smooth stromatolite morphologies at Shark Bay, one can expect that REY are also released from organic matter degradation into pore waters that eventually formed the stromatolitic carbonate. Indeed, REY normalization of the stromatolitic carbonate leachates to organic matter (Freslon et al. 2014) results in relatively flat REY distributions (Figure 6). Assuming qualitative REY substitution for calcium without REY fractionation into the carbonate crystal lattice, as suggested by Zhong and Mucci (1995) and Webb and Kamber (2000), this indicates a porewater control within the complex microbial mat systems on the REY geochemistry in Shark Bay stromatolite build‐ups as a result of organic matter degradation. However, no REY data on EPS are available, and the question remains open as to whether the REY signatures of organic matter in diverse aqueous systems (Freslon et al. 2014) are comparable to those of EPS in microbial mats.

In summary, our REY dataset supports the idea that the degradation of organic matter in a (semi)closed microbial mat system played a crucial role in shaping the REY geochemistry of stromatolitic carbonates at Shark Bay. Furthermore, the cerebroid textures of stromatolitic carbonates, which typically formed under the deepest water levels within the subtidal settings in the hypersaline Shark Bay lagoon (Figure 2, Jahnert and Collins 2012), show the most substantial chemical similarity to ambient water chemistry. One can speculate that this observation may be related to the least‐closed system conditions in subtidal cerebroid microbial mat build‐ups, which are constantly submerged and in contact with lagoon waters (Jahnert and Collins 2012; Suosaari, Reid, Playford, et al. 2016). In turn, stromatolite morphologies found in intertidal and supratidal settings are, at least temporarily, exposed and not continually flooded by lagoonal waters. The observation that deepest lagoonal waters most closely represent ambient water chemistry is further corroborated by observations from Miocene stromatolites of the Oberpullendorf Basin (Viehmann et al. 2023). Pure colloform stromatolites of the Oberpullendorf Basin indicate a basin restriction and do not show typical seawater‐like REY_SN_ patterns (Figure 4D) concomitant with a continuous depletion of bio‐essential elements in the carbonate leachates (Viehmann et al. 2023). In contrast, domal stromatolites show typical open‐ocean REY_SN_ patterns (Figure 4D) with elevated and constant bio‐essential element concentrations, indicating marine transgressions into the ancient lagoon (Viehmann et al. 2023). Thus, it appears from the Shark Bay and Oberpullendorf sample sites that stromatolitic carbonates formed in the deepest depositional environments of lagoonal settings, i.e., cerebroid structures in Shark Bay or domal stromatolites in the Oberpullendorf Basin, most closely represent the chemistry of ambient waters. In contrast, stromatolitic carbonates that formed in more shallow depositional environments were at least temporarily emerged and not continuously in exchange with ambient lagoon waters. This results in more closed‐system conditions in the microbial mat, leading to a greater influence of porewater chemistry on REY signatures in the carbonates and a lesser influence of ambient lagoon waters.

### 5.3 Implications for the Applicability of REY in Stromatolitic Carbonates for Paleo‐Environmental Reconstructions

Stromatolitic carbonates in Phanerozoic strata mostly represent extreme depositional environments where microbial life occupied ecological niches with (e.g., Suosaari, Reid, Abreu Araujo, et al. 2016; Howard and Sheldon 2025) or without (e.g., Rishworth et al. 2017) metazoan grazing pressure. Precambrian seawater chemistry was much different than in the Phanerozoic and more favorable to form primary stromatolitic Ca‐Mg carbonates (e.g., Grotzinger 1990), ranging, for instance, from one of the oldest stromatolites of the > 3.35 Ga Strelley Pool Fm. in Australia (e.g., Van Kranendonk et al. 2002; Viehmann et al. 2020) over stromatolites of the 2.44 Ga Rooinekke Group (South Africa; Schier et al. 2018), to stromatolites of the 1 Ga Proterozoic Paranoá Group (Brazil, Viehmann et al. 2019). Furthermore, Si‐rich seawater chemistry in the Archean also favored the formation of primary to very early diagenetic alternating Si‐ and carbonate‐rich stromatolite structures of the ca. 2.95 Ga Pongola Supergroup (South Africa; e.g., Siahi et al. 2018) and of Steep Rock (Canada, Fralick and Riding 2015). Marine environments during the Archean and in many parts of the Proterozoic, and their ecological niches, were also much less contested relative to the Phanerozoic (Howard and Sheldon 2025). This provided a multitude of favorable environmental conditions for microbial life to thrive and evolve. However, only a small subset of sub‐recent stromatolites is somewhat comparable to Precambrian stromatolites based on morphological differences, but also depends on different carbonate accretion mechanisms (e.g., Bosak et al. 2013; Reid et al. 2024). Furthermore, sub‐recent stromatolitic carbonates are often found close to discharge areas of different fluids such as groundwaters (e.g., Johannesson et al. 2014; Zeyen et al. 2021) that—in addition to diverse microbial communities that are capable of creating micro‐environments—increase alkalinity and facilitate carbonate precipitation (e.g., Dupraz and Visscher 2005; Reid et al. 2024, and references therein). These fluids favoring stromatolite carbonate build‐ups thus represent not only ambient lake or marine waters but also porewaters in the microbial mats and represent mixtures of two or more fluids in different ratios, which are also strongly dependent on seasonal variations. These fluids show substantial chemical variability, as observed in Shark Bay, and it is reasonable that many sub‐recent and Phanerozoic stromatolites exhibit diverse REY chemistries that are not directly comparable to the environmental conditions and ambient water chemistries in which the stromatolitic carbonates precipitated over several hundred years. The influence of pore waters in a (semi)closed microbial mat ecosystem on carbonate geochemistry based on the availability and degradation of organic matter such as EPS within or on the surface of microbial mats that is observed in Shark Bay microbial build‐ups (e.g., Dupraz and Visscher 2005), may also have severe impact on REY geochemistry in stromatolitic carbonates through deep time.

Many REY studies published in the last 10 years have focused on REY chemistry and their behavior in freshwater systems and have shown that the formerly considered “typical” seawater REY patterns may also appear in freshwater systems (e.g., Johannesson et al. 1997; Merschel, Bau, and Dantas 2017; Merschel, Bau, Schmidt, et al. 2017). This observation strongly raises caution about the sole use of REY geochemistry as a paleo‐environmental proxy in stromatolitic carbonates to reconstruct physico‐chemical conditions in ancient microbial habitats. Other chemical sediments that show “typical” seawater‐like REY_SN_ signatures, such as banded iron formations, are primarily associated with marine sequences based on their stratigraphic relationships and regional geological setting (e.g., Smith and Viehmann 2025). Stromatolites, however, which may also have formed in a variety of fresh or brackish environments, are only dependent on depositional environments within the photic zone. Thus, it is mandatory not to rely solely on geochemical proxies of stromatolites and other microbialites for paleo‐environmental reconstructions, but also to include other indicators, when possible, such as field relationships. Additionally, the sample size of stromatolites (individual laminae vs. bulk rock) should be related to the geochemical proxy of interest and its residence time in aqueous and marine environments (cf. Stüeken et al. 2023).

This study further broadens our understanding of the use of REY distributions in stromatolites from extreme environments. It indicates that REY geochemistry in combination with stromatolite morphology can be directly linked to individual depositional environments and relative water depths within the depositional area. This observation from a natural system should serve as a basis for future interdisciplinary geo‐microbiology studies to better understand REY cycling and speciation in complex microbial mat systems. These studies should include the role of REY during the uptake and degradation of organic matter, such as EPS, REY cycling by diverse microbial consortia, and REY speciation related to micro‐environmental conditions across different levels of stromatolite‐forming microbial mats. This information is incremental toward answering the long‐standing question of why some modern and Precambrian samples show typical seawater‐like REY_SN_ patterns. In contrast, others do not, and it is unclear which process(es) are responsible for these differences. This knowledge is of utmost importance for the application of chemical elements and their isotopes to stromatolitic carbonates with different morphologies and should be a prime target for future studies to reconstruct microbial habitats through deep time.

## 6 Conclusions

We provide the first comprehensive REY data from stromatolitic carbonate leachates and ambient waters of the hypersaline Shark Bay lagoon, Australia. Stromatolite morphologies include colloform, cerebroid, pustular, and smooth textures, sampled in combination with ambient lagoon waters from subtidal, intertidal, and supratidal environments. These data allow us to further explore the applicability and reliability of REY in stromatolitic carbonates as a proxy for reconstructing paleo‐environments. Due to very low REY concentrations in the carbonates and severe interferences of the carbonate matrix on the REY series, we applied a matrix separation and REY preconcentration method to accurately determine REY concentrations in the carbonates. The REY signatures in stromatolitic carbonates, ambient lagoon water, and groundwater of Shark Bay are different from those of open ocean seawater. REY signatures of stromatolitic carbonates of all morphologies are also different from those of ambient waters but show similarities to those of organic matter. This observation may indicate that organic matter degradation and subsequent REY release had an impact on pore fluid chemistry within the (semi)closed microbial mat system that eventually formed the stromatolitic carbonates. The carbonates show a middle REY_SN_ enrichment relative to light REY_SN_ and, except for the colloform structures, also an enrichment relative to the heavy REY_SN_. These differences can thus be attributed to the lack of comparability between today's water chemistry and the fluid chemistry from which the carbonate precipitated in the complex microbial mat system over the last hundreds of years. Colloform structures have the lowest and most variable Kd_(stromatolite‐fluid)_ values throughout the REY series, potentially related to REY fractionation onto surface sites of EPS. Cerebroid structures that formed in the deepest marine environments of the lagoon have the highest (> 875) and most constant Kd_(stromatolite‐fluid)_ values throughout the REY series and may be the most reliable archives to represent ambient water chemistry. It appears that stromatolite morphologies that formed in the deepest marine environments are the most reliable geochemical archives for ambient water chemistry. Stromatolitic carbonates of morphologies that formed in more shallow water environments and are temporarily emerged are not in continuous exchange with ambient waters. They may have been more strongly affected by microbial mat porewater chemistry with additional water mixing of ground‐ or rainwater. Our results imply that REY geochemistry, in combination with stromatolite morphology, can serve as a prime archive for reconstructing microbial habitats through deep time. Caution, however, is warranted, particularly with respect to carbonates formed in shallow depositional environments, which may have been severely impacted by chemically variable detrital inputs as well as seasonally changing fluid chemistry related to organic matter degradation, tides, water‐level changes, and mixtures of seawater with meteoric or groundwater.

## Conflicts of Interest

The authors declare no conflicts of interest.

## Supporting information


**Figure S1:** Close‐up and thin‐section photographs of stromatolite build‐ups SB19‐1‐1 (A, C), SB19‐2‐1 (B, D), SB19‐1‐2x, SB‐1‐3b, SB19‐2‐2x, SB19‐1‐4x, and SB19‐3‐1 (E). (A) The lower portion of SB19‐1‐1 consists of a compact, non‐laminated cerebroid structure with some fenestrae. The upper, slightly greenish part of the same stromatolite has a coarse‐laminated colloform structure and contains more quartz grains relative to the lower part of the specimen. (B) Stromatolites with a cerebroid structure overgrow coarse‐grained substrate. Shell fragments and abundant quartz grains are found in the substrate; the cerebroid stromatolite consists of dense aragonite cement, partly intervened with fenestrae structures. (C, D) Newly, with a microdrill, sampled individual parts of specimens SB19‐1‐1 and SB19‐2‐1. (E) Samples SB19‐1‐2x (intertidal, colloform), SB‐1‐3b (intertidal, pustular), SB19‐2‐2x (subtidal, cerebroid), SB19‐1‐4x (intertidal, colloform), and SB19‐3‐1 (supratidal, smooth/pavement) that are taken as homogenous sample powders from Martin et al. (2023).
**Figure S2:** REY_SN_ patterns of carbonate (A) and water (B) CRMs of this study relative to published data. (A) JDo‐1 REY concentrations of our carbonate leaching approach are 44% ± 5% lower relative to complete digestion data published by Dulski (2001). The REY_SN_ patterns are sub‐parallel, indicating that no REY fractionation occurred during our carbonate leaching procedure. (B) River (SLRS‐6) and seawater (NASS‐7) CRM data obtained in this study by a seafast SP2 system coupled to an iCAP‐TQ‐ICPMS are in good agreement with published data and overlap with data of Ebeling et al. (2022).

**Table S1:** Matrix element concentrations and δ^13^c‐δ^18^o values of Shark Bay stromatolites.

**Table S2:** REY concentrations of CRMs relative to published data.

**Table S3:** REY & Ca concentrations and Kd values between stromatolite and waters.

## Data Availability

All data that support the findings of this study are available within the tables of this publication and are also available upon request from the corresponding author.
