# Supplementary material for: Contrasting Rare Earth Element Signatures Between Stromatolitic Carbonates and Lagoon Waters at Shark Bay, Western Australia: Implications for Paleo‐Environmental Reconstructions of Microbial Habitats
Source: Geobiology. 2026 Jun 10;24(3):e70055. doi: 10.1111/gbi.70055 (PMC13250952; doi:10.1111/gbi.70055)
Supplement: Supplementary file 1 — Figure S1: Close‐up and thin‐section photographs of stromatolite build‐ups SB19‐1‐1 (A, C), SB19‐2‐1 (B, D), SB19‐1‐2x, SB‐1‐3b, SB19‐2‐2x, SB19‐1‐4x, and SB19‐3‐1 (E). (A) The lower portion of SB19‐1‐1 consists of a compact, non‐laminated cerebroid structure with some fenestrae. The upper, slightly greenish part of the same stromatolite has a coarse‐laminated colloform structure and contains more quartz grains relative to the lower part of the specimen. (B) Stromatolites with a cerebroid structure overgrow coarse‐grained substrate. Shell fragments and abundant quartz grains are found in the substrate; the cerebroid stromatolite consists of dense aragonite cement, partly intervened with fenestrae structures. (C, D) Newly, with a microdrill, sampled individual parts of specimens SB19‐1‐1 and SB19‐2‐1. (E) Samples SB19‐1‐2x (intertidal, colloform), SB‐1‐3b (intertidal, pustular), SB19‐2‐2x (subtidal, cerebroid), SB19‐1‐4x (intertidal, colloform), and SB19‐3‐1 (supratidal, smooth/pavement) that are taken as homogenous sample powders from Martin et al. (2023). Figure S2: REYSN patterns of carbonate (A) and water (B) CRMs of this study relative to published data. (A) JDo‐1 REY concentrations of our carbonate leaching approach are 44% ± 5% lower relative to complete digestion data published by Dulski (2001). The REYSN patterns are sub‐parallel, indicating that no REY fractionation occurred during our carbonate leaching procedure. (B) River (SLRS‐6) and seawater (NASS‐7) CRM data obtained in this study by a seafast SP2 system coupled to an iCAP‐TQ‐ICPMS are in good agreement with published data and overlap with data of Ebeling et al. (2022). [file GBI-24-e70055-s002.docx]

**Supplemental information to**

**Contrasting rare earth element signatures between stromatolitic carbonates and lagoon waters at Shark Bay, Western Australia: implications for paleo-environmental reconstructions of microbial habitats**

**Supplemental figures**


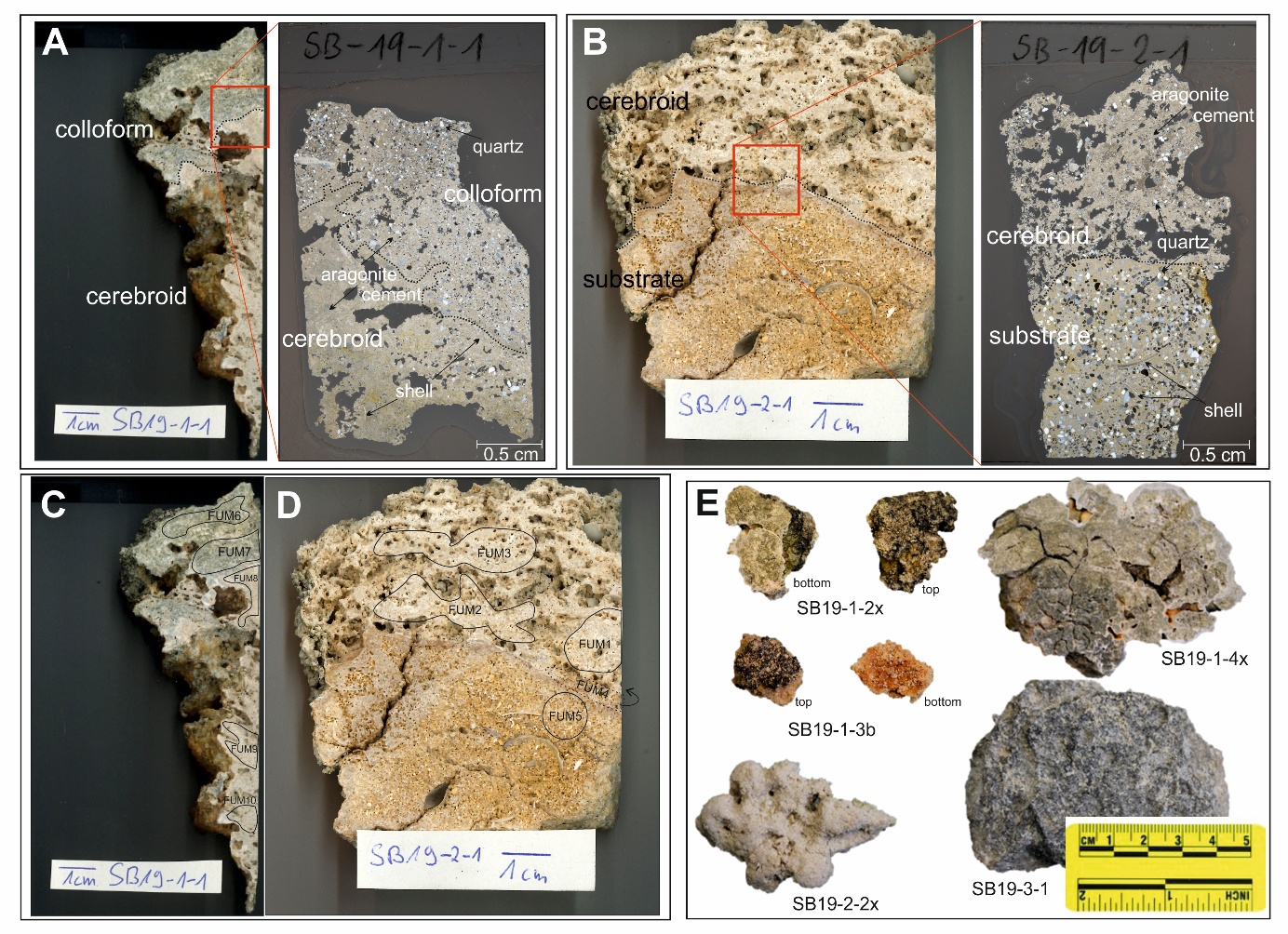


**Figure S1:** Close-up and thin-section photographs of stromatolite build-ups SB19-1-1 (A, C), SB19-2-1 (B, D), SB19-1-2x, SB-1-3b, SB19-2-2x, SB19-1-4x, and SB19-3-1 (E). (A) The lower portion of SB19-1-1 consists of a compact, non-laminated cerebroid structure with some fenestrae. The upper, slightly greenish part of the same stromatolite has a coarse-laminated colloform structure and contains more quartz grains relative to the lower part of the specimen. (B) Stromatolites with a cerebroid structure overgrow coarse-grained substrate. Shell fragments and abundant quartz grains are found in the substrate; the cerebroid stromatolite consists of dense aragonite cement, partly intervened with fenestrae structures. (C, D) Newly, with a microdrill, sampled individual parts of specimens SB19-1-1 and SB19-2-1. (E) Samples SB19-1-2x (intertidal, colloform), SB-1-3b (intertidal, pustular), SB19-2-2x (subtidal, cerebroid), SB19-1-4x (intertidal, colloform), and SB19-3-1 (supratidal, smooth/pavement) that are taken as homogenous sample powders from Martin et al. (2023).


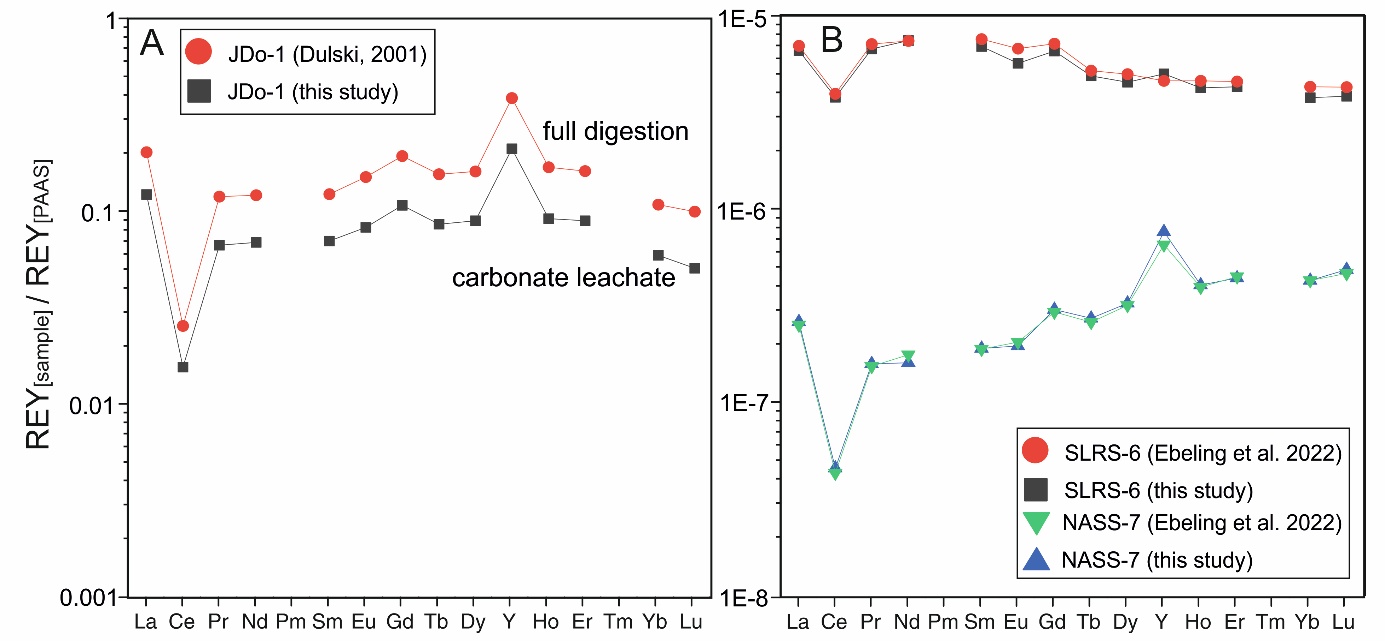


**Figure S2:** REY_SN_ patterns of carbonate (A) and water (B) CRMs of this study relative to published data. (A) JDo-1 REY concentrations of our carbonate leaching approach are 44 ±5 % lower relative to complete digestion data published by Dulski (2001). The REY_SN_ patterns are sub-parallel, indicating that no REY fractionation occurred during our carbonate leaching procedure. (B) River (SLRS-6) and seawater (NASS-7) CRM data obtained in this study by a seafast SP2 system coupled to an iCAP-TQ-ICPMS are in good agreement with published data and overlap with data of Ebeling et al. (2022).

**Supplemental references**

Dulski, P., 2001. Reference materials for geochemical studies: New analytical data by ICP-MS and critical discussion of reference values. *Geostand. Newsl.* **25**, 87–125.

Ebeling A., Zimmermann T., Klein O., Irrgeher J., Pröfrock D., 2022. Analysis of seventeen certified water reference materials for trace and technology-critical elements. *Geostand. Newsl.* **46**, 351-378.

Martin A.N., Markowska M., Chivas A.R., Weyer S, 2023. Assessing the reliability of modern marine stromatolites as archives for the uranium isotope paleoredox proxy. *Geochimica et Cosmochimica Acta* **345**, 75–89.
